# A pilot study of peripheral blood DNA methylation models as predictors of knee osteoarthritis radiographic progression: data from the Osteoarthritis Initiative (OAI)

**DOI:** 10.1038/s41598-019-53298-9

**Published:** 2019-11-14

**Authors:** Christopher M. Dunn, Michael C. Nevitt, John A. Lynch, Matlock A. Jeffries

**Affiliations:** 1 University of Oklahoma Health Sciences Center, Department of Internal Medicine, Division of Rheumatology, Immunology, and Allergy, Oklahoma City, OK USA; 2 Oklahoma Medical Research Foundation, Arthritis and Clinical Immunology Program, Oklahoma City, OK USA; 3 University of California San Francisco, San Francisco, CA USA

**Keywords:** Prognostic markers, Osteoarthritis

## Abstract

Knee osteoarthritis (OA) is a leading cause of chronic disability worldwide, but no diagnostic or prognostic biomarkers are available. Increasing evidence supports epigenetic dysregulation as a contributor to OA pathogenesis. In this pilot study, we investigated epigenetic patterns in peripheral blood mononuclear cells (PBMCs) as models to predict future radiographic progression in OA patients enrolled in the longitudinal Osteoarthritis Initiative (OAI) study. PBMC DNA was analyzed from baseline OAI visits in 58 future radiographic progressors (joint space narrowing at 24 months, sustained at 48 months) compared to 58 non-progressors. DNA methylation was quantified via Illumina microarrays and beta- and M-values were used to generate linear classification models. Data were randomly split into a 60% development and 40% validation subsets, models developed and tested, and cross-validated in a total of 40 cycles. M-value based models outperformed beta-value based models (ROC-AUC 0.81 ± 0.01 vs. 0.73 ± 0.02, mean ± SEM, comparison p = 0.002), with a mean classification accuracy of 73 ± 1% (mean ± SEM) for M- and 69 ± 1% for beta-based models. Adjusting for covariates did not significantly alter model performance. Our findings suggest that PBMC DNA methylation-based models may be useful as biomarkers of OA progression and warrant additional evaluation in larger patient cohorts.

## Introduction

Osteoarthritis (OA) is the leading cause of chronic disability in the United States, and is the third most rapidly rising chronic medical condition associated with disability worldwide^1,2^. Despite its importance and economic impact, there are no disease-modifying anti-osteoarthritic drugs (DMOADs) approved by the US Food and Drug Administration (FDA) or European Medicines Agency, in stark contrast to the multitude of biologic and nonbiologic disease-modifying treatments available in other forms of arthritis^3^. This lag in the development of DMOADs is due in no small part to a lack of easily accessible radiographic and/or biochemical biomarkers to diagnose OA and discriminate patient phenotypes, including prediction of future progressors.

Accordingly, much attention has recently been focused on the development of diagnostic and prognostic biomarkers for OA. Several groups have described biomarker candidates for OA diagnosis, discriminating OA patients from healthy controls based on peripheral blood analytes. In 2014, Ramos *et al*. described an mRNA-based peripheral blood signature which could discriminate OA patients from matched controls^4^, with a c-statistic of 0.97. In 2018, Li *et al*. published a reanalysis of these data using a different machine learning technique that performed at an equally high sensitivity and specificity on a reduced subset genes^5^. In 2018, Huang and colleagues used data and biospecimens from the DOXY (doxycycline for treatment of OA) clinical trial to examine four plasma biomarkers^6^, and found baseline levels of lipopolysaccharide binding protein (LBP) were associated with future radiographic progression (TIC over 18 months OR = 1.418).

A more difficult but perhaps more clinically relevant biomarker task, however, is the classification of OA patients into distinct phenotypes. The largest study yet to take on the task of OA phenotype discrimination was an analysis of serum and urine biochemical biomarkers performed by the Foundation for the National Institutes of Health (FNIH) OA Biomarkers Consortium (OABC-FNIH), using data and biospecimens from the longitudinal, US-based Osteoarthritis Initiative (OAI) study^7,8^. In this study, the authors produced a model combining three serum and urine biomarkers that could discriminate radiographic and pain progressors from nonprogressors with a receiver operator characteristic area under the curve (AUC-ROC, c-statistic) of 0.631^9^. Baseline values of these parameters did not offer substantial predictive capability; rather, models utilized a time-integrated concentration (TIC) approach tracking analyte measurements over a period of years.

A drawback of traditional biochemical biomarkers such as those used in the OABC-FNIH is their inherent variability. Epigenetic assays offer theoretical advantages as biomarkers. Most notably, an epigenetics-based assay has the potential to offer prognostic information based on a single time point reflecting early, relatively stable gene regulatory changes that precede gene transcription and subsequent protein translation. Early epigenetic changes in peripheral blood have been found to be useful biomarkers in several rheumatic diseases including rheumatoid arthritis^10^ and systemic lupus erythematosus^11^, as well as several chronic low-level inflammatory diseases, including type-2 diabetes mellitus risk^12^ and cardiovascular disease^13^. Several recent studies, including our own, have demonstrated alterations in joint tissue epigenetic patterns associated with OA development and progression^14–18^; however, no analyses of blood epigenetic changes have yet been published.

In this pilot study, we aimed to evaluate the potential of peripheral blood mononuclear cell (PBMC)-based epigenetic models to predict future knee OA radiographic progression in a well-matched cohort of patients from the Osteoarthritis Initiative (OAI), using similar definitions to those previously published in OABC-FNIH studies^9^.

## Results

### Baseline patient demographic, disease, and PBMC composition characteristics were well matched. Models developed using only patient characteristics were not predictive of future progression

We first identified a group of 58 OA radiographic progressors with baseline Kellergren-Lawrence (K/L) radiographic grade 2–3, symptomatic knee OA who exhibited ≥0.7 mm of joint space width (JSW) loss over the first 24 months of follow-up and persistent JSW loss at 48 months from the longitudinal, US-based Osteoarthritis Initiative (OAI) study. We then matched 58 nonprogressors with ≤0.5 mm of joint space width loss over 48 months of follow-up by age category, sex, BMI category, ethnicity, and baseline KL radiographic grade (Table 1, Supplementary Table 1). Although not included in our matching criteria, there were no statistical differences in baseline JSW, NSAID use, or smoking history between the two groups. We did note a statistically significant increase in baseline Western Ontario and McMaster (WOMAC) pain subscale among progressors (21.5 ± 2 mean ± SEM vs. 17.0 ± 1.5 points on a 0–100 point normalized scale, P = 0.05).

**Table 1:** Patient group characteristics.

	Radiographic progressors (cases) (n = 58), mean ± SEM	Nonprogressors (controls) (n = 58), mean ± SEM	2-tailed P value
**Baseline characteristics**			
Age	60 ± 1	60 ± 1	0.90
Sex (% female)	53.4%	60.3%	0.13
BMI	30.5 ± 0.5	30.9 ± 0.6	0.61
Ethnicity (% caucasian)	88%	88%	1.00
Smoking (% positive)	46%	43%	0.71
NSAID use (% positive)	29%	17%	0.13
*Mean WOMAC pain (0–100 point normalized scale)*	*21*.*5 ± 2*	*17*.*0 ± 1*.*5*	*0*.*05*
Mean JSW (mm)	3.9 ± 0.1	4.0 ± 0.1	0.34
Baseline K/L grade 2	25	35	0.09
Baseline K/L grade 3	33	23	
**Baseline estimated PBMC composition**			
CD8 + T cells	8.5 ± 0.7%	8.6 ± 0.6%	0.9
CD4 + T cells	21 ± 0.9%	21 ± 1%	0.9
NK cells	7.4 ± 0.5%	7.6 ± 0.6%	0.9
B cells	9.6 ± 0.5%	9.8 ± 0.5%	0.8
Monocytes	7.4 ± 0.3%	6.7 ± 0.3%	0.12
Granulocytes	51 ± 1%	51 ± 1%	0.9
**Baseline comorbidities**			
Type 2 diabetes	4 (patients/58)	6 (# patients/58)	0.74
History of heart attack	0	0	n/a
History of heart failure	2	3	1.0
History of stroke	2	4	0.68
History of lung disease	0	0	n/a
History of cancer	1	4	0.36

**Figure 1:**
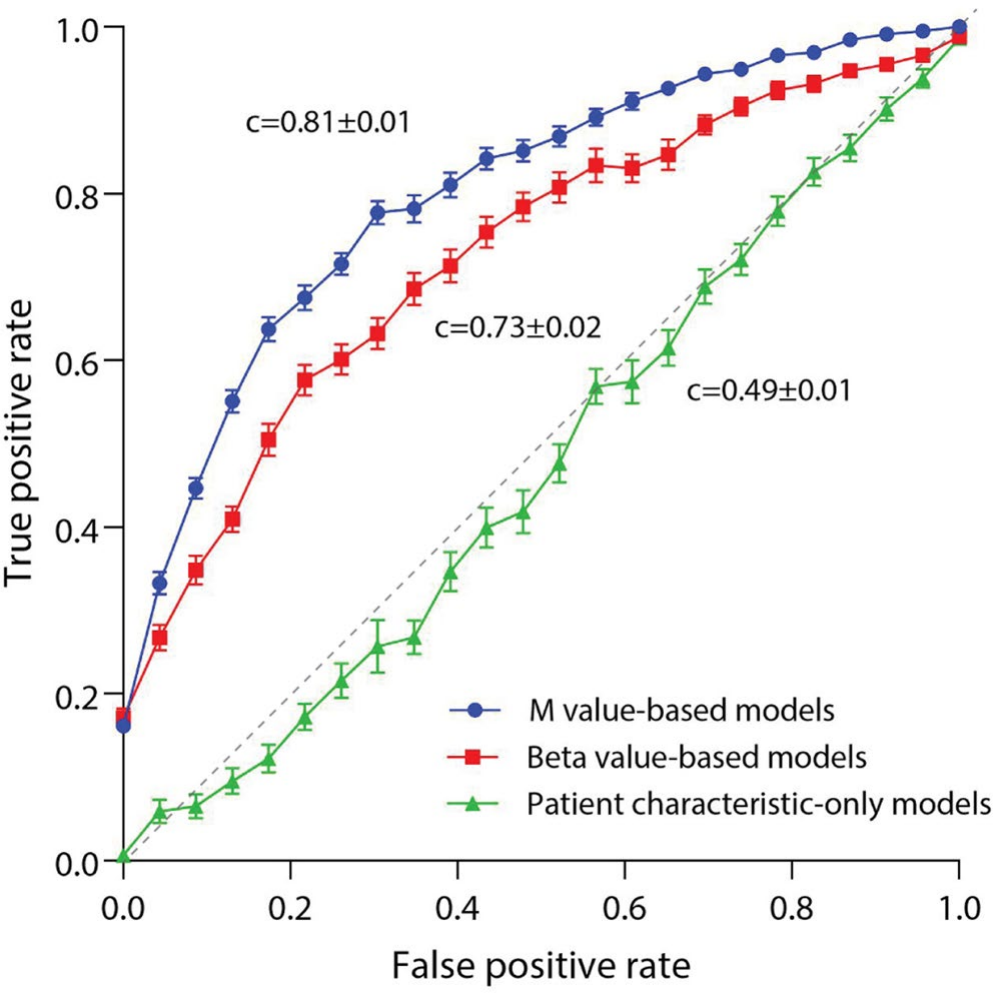
Mean receiver operator characteristic (ROC) curves for patient characteristic-only models, Beta value-based models, and M value-based models when tested on unseen (lockbox) data. Curves represent mean values over 40 cycles of development, error bars represent SEM. ROC-AUC (c-statistic) values are given as mean ± SEM.

Recent studies have reported that clinical characteristics alone can be used to model future OA progression^19^. To ensure that our models were not being affected by patient characteristics, we first developed models using baseline patient characteristics data alone, without including DNA methylation data. These models included age, sex, BMI, baseline JSW, baseline WOMAC pain, smoking history, and NSAID use. Baseline models were not able to discriminate the two groups (receiver operator characteristic area under the curve ROC-AUC, c-statistic = 0.49 ± 0.01 mean ± SEM, accuracy = 50 ± 0.7%) (Fig. 1). Although infrequent, we then added data regarding comorbidities to these baseline models, including history of heart attack, heart failure, diabetes, lung disease, and cancer (Table 1). This did not improve the discriminatory capability of baseline patient characteristic models (p = 0.28 for comparison with models not including comorbidity data).

DNA methylation data from mixed PBMC samples can be skewed by underlying differences in cellular composition among groups^20^. As the OAI dataset does not include data on individual blood sample cellular composition, we estimated this using a computational approach^21^. There were no statistically significant differences in estimated PBMC composition between the two groups; however, we did note a trend towards increased monocyte counts in cases (progressors 7.4 ± 0.3% mean ± SEM vs. nonprogressors 6.7 ± 0.3%, P = 0.12) (Table 1). Accordingly, we corrected our dataset for PBMC composition differences using frozen surrogate variable analysis (FSVA), a technique previously demonstrated to be robust in correcting cellular composition differences in genome-wide DNA methylation data^22,23^, before developing our epigenetic models.

### Models developed based on PBMC DNA methylation data are predictive of radiographic progression

**Figure 2:**
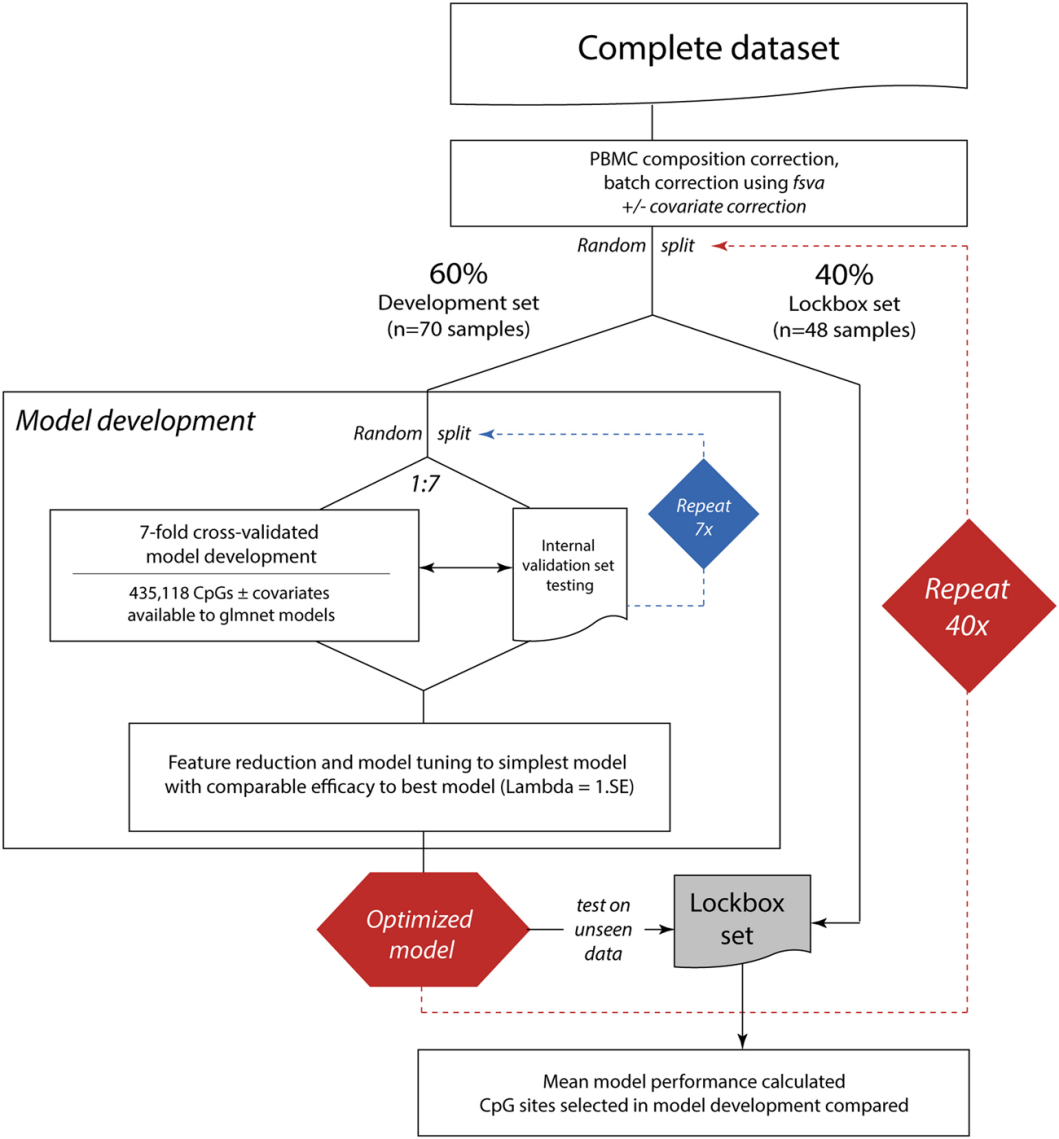
OA rapid progressor PBMC DNA methylation-based machine learning discriminant model development plan.

Traditionally, epigenome-wide association studies have reported DNA methylation data as beta values, defined as the fraction methylation (0–1 scale) of each CpG site included in the array. However, beta values are characterized by high heteroscedasticity (most beta values fall within extreme high- and low-percent methylation levels). Therefore, we also analyzed M values (the log_2_ ratio of methylated:unmethylated probe intensities for a given CpG site), which are approximately homoscedastic, in our models^24^. In our analysis, models based on PBMC DNA methylation data were consistently capable of discriminating those patients who would go on to experience radiographic progression from nonprogressors (Figs 1, 2, Table 2). Models based on M values outperformed those based on beta values (M value models c = 0.81 ± 0.01, mean ± SEM, vs. beta value models 0.75 ± 0.01, comparison p = 0.002), with corresponding accuracies of 73 ± 1%, mean ± SEM, for M and 69 ± 1% for beta based models. The mean number of CpGs selected for inclusion during model development and optimization was 22 ± 2 (mean ± SEM) for M-based and 19 ± 2 for beta value-based assays **(**Supplementary Fig. 1**)**, this was not statistically significantly different between the two DNA methylation measures (p = 0.32).

**Table 2:** Performance of PBMC DNA methylation models to predict future radiographic progression in OA patients when evaluating previously unseen data.

	M-value based models (mean ± SEM)	Beta value-based models (mean ± SEM)
ROC-AUC (c-statistic)	0.81 ± 0.01	0.75 ± 0.01
Accuracy	73 ± 1%	69 ± 1%
Odds ratio	11 ± 2	9 ± 2
Sensitivity	74 ± 1%	73 ± 2%
Specificity	70 ± 1%	70 ± 1%

We then added patient characteristic covariate data to model development, including age, sex, BMI, baseline JSW, baseline WOMAC pain, smoking history, and NSAID use. Including these data did not alter model performance (M value models c = 0.80 ± 0.01, beta value models c = 0.78 ± 0.01, both nonsignificant compared to models without covariates). Finally, as our analysis included data from two methylation assay types, we then analyzed model performance for 450k and 850k chips separately; both types demonstrated equivalent performance in our models (p = 0.27).

### A subset of DNA methylation CpG sites were selected frequently in multiple rounds of model development and were enriched in functional pathways previously associated with OA development

Although 969 CpGs were selected in at least one round of model development in either M or Beta based models, a subset of CpG sites were selected several times (Table 3, complete list of CpGs selected in Supplementary Table 2). Many of these most frequently selected CpGs in both M value-based and Beta value-based model development were shared; for example, 7 of the top 20 most frequently selected CpGs in both M- and Beta models were identical. The majority of the top 20 CpGs selected during modeling were associated with CpG islands (13 of 20 M- and 16 of 20 Beta-based models), including all but one of the CpG sites shared among the two methylation measures, suggesting these sites may play a role in gene regulation^25^.

**Table 3:** Top 20 CpGs selected for supervised model development. CpGs shared by both Beta-value-based and M-value-based models are highlighted in bold (n = 7).

M value-based algorithm feature	# of development rounds selected (out of 40)	Associated gene	CpG location (regulatory region)	Location within CpG island	Beta value-based algorithm feature	# of development rounds selected (out of 40)	Associated gene	CpG location (regulatory region)	Location within CpG island
cg15974085	15	*C18orf55; FBXO15*	TSS200	Island	**cg11865413**	**15**			**N_Shelf**
**cg26384229**	**14**	***ALG10B***	**TSS200**	**Island**	**cg21643086**	**15**			
**cg21643086**	**12**				**cg17745251**	**11**	***C10orf140***	**Body**	**Island**
**cg03687650**	**11**	***OSBPL5***	**Body**	**S_Shelf**	**cg06409741**	**11**	***RASA3***	**Body**	**Island**
**cg06409741**	**11**	***RASA3***	**Body**	**Island**	**cg03870777**	**10**	***KRT18***	**TSS200**	**N_Shore**
cg18111500	10				cg17956079	10	*PLEKHB1*	TSS200	
cg07772660	9	*MST1P9*	Body	Island	**cg26384229**	**9**	***ALG10B***	**TSS200**	**Island**
**cg11865413**	**9**			**N_Shelf**	**cg03687650**	**7**	***OSBPL5***	**Body**	**S_Shelf**
cg03212634	9				cg10306485	7	*RASA3*	Body	S_Shelf
cg00142933	8	*LIMS2*	Body	N_Shore	cg19559392	7	*MORN2; DHX57*	5′UTR; TSS1500	S_Shore
cg16001460	8	*PRIM2*	TSS1500		cg05587853	7	*MSL2*	TSS1500	Island
**cg17745251**	**7**	***C10orf140***	**Body**	**Island**	cg14616423	7			
cg14330460	7				cg08728848	6			
**cg03870777**	**6**	***KRT18***	**TSS200**	**N_Shore**	cg07379140	5	*ALG14*	TSS200	Island
cg02215141	6			N_Shore	cg02962630	5	*DLL4*	Body	Island
cg20200361	6	*THADA*	Body		cg16790849	5	*NEU1*	Body	N_Shore
cg07258847	6				cg00143249	5	*MNX1*	Body	Island
cg23226134	5	*CLCN6;MTHFR*	1st Exon	Island	cg10609068	4	*ANO6 PLEKHA9*	TSS1500; 5′UTR	N_Shore
cg16121685	5	*UHMK1*	TSS200	Island	cg14710040	4	*EEF2*	TSS200	Island
cg10966582	5	*MST1P9*	Body	Island	cg17310773	4	*SLC25A17*	1stExon	Island

Although we did not perform a differential methylation analysis in the traditional sense, we reasoned that CpG sites chosen for inclusion in our models might still cluster in functional pathways important in OA. To investigate this, we performed gene ontology analysis using the Ingenuity Pathway Analysis (IPA) system of genes associated with CpG sites chosen in at least one round of model development. The most significantly enriched canonical pathways enriched in this gene set included the antigen presentation pathway (n = 6 genes, p = 7E-4), adenosine monophosphate kinase (AMPK) signaling (n = 15, p = 1E-3, and sonic hedgehog signaling (n = 4 genes, p = 9E-3), among others (Table 4). The top upstream regulators identified by IPA include the transcription factor PITX2 (n = 14 genes, p = 1E-5), histone H3 (n = 29 genes, p = 7E-5) and H4 (n = 14,p = 9.6E-5), miR-141 (n = 9, p = 1.9E-4), miR-9 (n = 5, p = 3.4E-4), miR-137 (n = 2, p = 8.2E-4), and bone morphogenic protein 2 (BMP2, n = 15, p = 9.0E-4), a TGF-β superfamily member, among others (Table 4).

**Table 4:** Ontology analysis of genes associated with rapid OA progressor DNA methylation models.

Canonical pathway	p-value	Genes associated	
Antigen presentation	7.08E-04	*CIITA*,*HLA-DPA1*,*HLA-DPB1*,*PSMB8*,*PSMB9*,*TAP2*	
AMPK signaling	1.05E-03	*AK8*,*CFTR*,*CHRM2*,*CHRNA2*,*CHRNA9*,*EEF2*,*FOXG1*,*INS*,*PDPK1*,*PPM1E*,*PRKACB*,*RAB7A*,*RPTOR*,*SMARCA4*,*SMARCD3*	
Sonic hedgehog signaling	9.33E-03	*GLIS1*,*GLIS2*,*PRKACB*,*SUFU*	
Synaptogenesis Signaling Pathway	1.58E-02	*AP2A2*,*CACNB4*,*CDH22*,*CDH23*,*CDH4*,*EIF4EBP3*,*EPHA8*,*EPHB3*,*FGR*,*GRIN2B*,*PRKACB*,*SHC2*,*STX1B*,*SYN2*,*SYT15*,*SYT5*	
Aryl Hydrocarbon Receptor Signaling	1.95E-02	*ALDH1A3*,*ARNT*,*CDK6*,*CDKN2A*,*NCOA2*, *NR2F1*,*RXRG*,*SMARCA4*,*TP73*	
Endocannabinoid Neuronal Synapse Pathway	2.75E-02	*CACNB4*,*CACNG3*,*CACNG8*,*CNR2*,*GNG7*,*GRIN2B*,*PRKACB*,*PTGS2*	
Autophagy	2.82E-02	*ATG7*,*CTSO*,*LAMP1*,*SQSTM1*,*WIPI1*	
**Upstream regulator**	**Molecule type**	**p-value**	**Number of associated genes**
PITX2	transcription regulator	1.34E-05	14
Bvht	long noncoding RNA	5.48E-05	10
histone H3	group	6.93E-05	29
histone H4	group	9.63E-05	14
miR-141	microRNA	1.91E-04	9
ASCL1	transcription regulator	3.09E-04	10
miR-9	microRNA	3.37E-04	5
LHX6	transcription regulator	4.42E-04	5
miR-137	microRNA	8.23E-04	2
BMP2	growth factor	8.96E-04	15

## Discussion

Biomarkers hold great potential for improving clinical outcomes in common chronic diseases including OA. The early identification of OA patients who are likely to progress radiographically, clinically, or both, will allow for the enrichment of clinical trials of disease-modifying antiosteoarthritic drugs (DMOADs) with appropriate patients. Biomarkers of progression may also allow clinicians to stratify early OA patients, offering personalized care by taking into account an individual’s likelihood to respond to a particular therapy. In this pilot study, we sought to test whether baseline peripheral blood mononuclear cell DNA methylation data could be used to develop models to discriminate those patients who would go on to experience radiographic progression from nonprogressors. While based on a relatively small number of patients from a single cohort, our results nonetheless suggest that peripheral blood-based epigenetic models may be useful for OA subtype discrimination and should be evaluated in future larger studies of heterogeneous OA patients from additional cohorts.

Several groups have previously investigated biomarkers predictive of future OA progression. The largest biochemical study in this regard was the OAI biomarker studies including the osteoarthritis biomarker consortium of the Foundation for the National Institutes of Health (OABC-FNIH) which investigated a targeted set of biochemical biomarkers as predictors of OA pain and radiographic progression. Their analysis identified a combination of three biochemical biomarkers for discrimination of pain and radiographic progression from nonprogressors with a c-statistic of 0.631^9^, although they used a time-integrated concentration (TIC) approach, requiring multiple biomarker measurements over time as input into their prognostic model. This same study also evaluated the predictive capability of baseline biomarkers alone, but these not demonstrate robust differentiation of progressors (c = 0.586)^9^.

Radiographic biomarkers predicting future disease progression have also been evaluated. In 2016, Collins *et al*. reported models based on changes in MRI semi-quantitative markers over 24 months to predict the likelihood of radiographic progression at 48 months as part of the OABC-FNIH study^26^; the best model achieved a c-statistic of 0.74. Like the OABC-FNIH biochemical biomarker study, this study leveraged changes in imaging parameters over time to predict future progression. Trabecular bone texture (TBT) is perhaps the best-studied single imaging biomarker for prediction of future OA progression. Kraus *et al*. evaluated this in FNIH patients, with a best composite model using baseline TBT to predict future radiographic progression with a c-statistic of 0.624^27^. This is somewhat lower than previous reports using TBT, Janvier and colleagues, for example, reported a c-statistic of AUC 0.77 in 2017^28^.

There have also been recent reports using machine learning to model future progression based on combinations of baseline imaging and demographic data. Joseph and colleagues in 2018 published the results of three such models, with a maximum c-statistic of 0.72 to predict progression to moderate-severe OA over 8 years including demographic data, risk factors, K/L score, cartilage WORMS score, meniscal tear, and cartilage MRI T2 imaging data^29^ from the OAI cohort. Also in 2018, Halilaj and colleagues published the results of a mixed-effects mixture model within the OAI cohort to predict joint space narrowing in 1243 subjects. They described an impressive c-statistic of 0.86 for prediction of future progression using a combination of baseline and year 1 follow-up variables, using a 10-cycle, 90% development/10% lockbox validation data splitting approach with 10-fold internal cross-validation, not unlike our methodology. Models based solely on baseline data, however, did not predict future radiographic progression (c ≤ 0.6)^30^.

Several recent studies have also identified mitochondrial haploptypes as biomarkers which can discriminate OA phenotypes. For example, in 2012 Fernandez-Moreno *et al*. identified significant increases in cartilage-specific biomarkers of OA in patients carrying mitochondrial haplogroup H associated with the onset of OA^31^. Similarly, this group later identified a decrease in incident OA risk in patients with mitochondrial haplogroup J compared to hpalogroup H (HR 0.7, p < 0.05) in both the OAI and CHECK cohorts^32^, as well as slowing of the rates of radiographic progression rate among OA patients with haplogroup T compared to other haplogroups within the OAI cohort^33^. Future studies should investigate potential complementarity between mitochondrial haplogroups and DNA methylation as predictive OA biomarkers.

Much work has been done over the past several years linking epigenetic changes, particularly DNA methylation, with OA. Our group and others have noted differential DNA methylation in inflammatory cell pathways in both cartilage and subchondral bone from OA patients^14–17,34,35^. A subgroup of end-stage OA patients’ cartilage demonstrates a fingerprint of epigenetic changes within inflammatory genes^18,36^. Given chondrocyte and subchondral bone DNA methylation aberrancy in both knee and hip OA, along with the known contributions of chronic inflammation and inflammatory gene epigenetic changes to OA, the DNA methylation patterns within PBMCs associated with OA progression we describe in this report are intuitively consistent with previously published data.

Our PBMC DNA methylation-based approach has a few potential advantages over previously published biomarkers. Most notably, our approach offers predictive capability based on a single baseline blood draw. Further, our models demonstrated higher c-statistic values than previously described biochemical biomarkers, although this should be interpreted cautiously given our relatively small group sample size and lack of non-OAI cohort confirmation. We also performed a traditional DNA methylation analysis of progressors and nonprogressors and did not identify significantly differentially methylated positions (DMPs) with a false data rate-corrected p-value ≤ 0.05. This is not particularly surprising given our mixed PBMC population. Our approach utilized the combined effects of multiple CpG sites when developing DNA methylation-based predictive models.

Relatively few of the CpG sites selected most frequently during model development have been previously associated with OA. A CpG near the transcription start site of *KRT18* was selected in both M- and Beta-model development. Reductions in KRT18 expression have been associated with lumbar spine OA^37^. *LIMS2*, selected in M-development, is a focal adhesion molecule differentially expressed in cartilage and subchondral bone of frizzled-related protein (Frzb) knockout mice^38^. A CpG site selected in M-based assay development was located near *CLCN6*, which has been associated with life expectancy and aging^39^. This same CpG site is also associated with the gene *MTHFR*, which is a rheumatoid arthritis (RA) risk allele^40^, and the protein encoded by *MTHFR* is the target of the RA disease-modifying antirheumatic drug methotrexate. Only one published association has been found linking *MTHFR* gene mutations and OA of the knee, hand, and hip in a Turkish population^41^. A CpG within *DLL4* was selected in Beta-based models. Dll4, a component of the Notch pathway, has been previously associated with the development of OA of the temporomandibular joint^42^. *EEF2*, also selected during Beta-based model development, has been shown to play a role in intervertebral disc degeneration and is the target of the OA-associated microRNA-143-5p^43^. This paucity of previously published OA association in model-selected CpGs is not surprising, as our study is the first report of peripheral blood epigenetic patterns in OA.

Despite the lack of previous OA associations among the specific CpG sites included in our models, gene ontology analysis revealed several canonical gene pathways and upstream regulators that have been previously described in OA. Unfortunately, there have not been previous epigenetic analyses of peripheral blood cells in OA in humans or mice to compare our data to. However, many pathways and regulators previously described in articular tissues overlap with our findings. For example, AMP kinase (AMPK) deficiency in chondrocytes accelerates both age- and trauma-associated OA in mice^44^, whereas a pharmacological stimulator of AMPK attenuates post-traumatic OA in rats^45^. The endocannabinoid system within synovial tissue has been associated with OA pain^46^. Autophagy has been widely associated with OA. Age-related loss of autophagy has been linked with OA severity in human^47^ and murine^48^ joint tissue. Furthermore, cartilage-specific deletion of mammalian target of rapamycin (mTOR) increases autophagy signaling and protects against post-traumatic OA induction in mic^49^. Among the topstream regulators identified in our ontology analysis was PITX2, a component of the Wnt signaling pathway key to mensenchymal stem cell function which is altered in OA patients^50^. MicroRNA-141 is dysregulated in human OA and contributes to pathogenesis by augmentation of lipid metabolism^51^, and is important in maintaining the appropriate expression of key osteoblast differentiation proteins including Runx2, Sclerostin, ALP, and Dlx5^52^. MicroRNA-9 has been shown to directly augment the expression of the key catabolic enzyme matrix metalloproteinase-13 (MMP13) in OA patients^53^ and animal models^54^. MicroRNA-137 regulates chondrocyte metabolism via targeting Runx2^55^. Finally, bone morphogenic protein-2 (BMP2) upregulation has been linked to OA development and increased matrix turnover^56^. It is a key regulator of chondrocyte metabolism^57^ and has been suggested as a novel OA therapeutic^58^. Future studies of circulating inflammatory cells and cell subsets in OA patients and/or OA animal models will be critical to confirmation of our findings and will help elucidate whether common pathway deficiencies, i.e. autophagy or Wnt signaling, are defective globally in OA rather than acting in a jont-specific manner.

Our study does have several weaknesses, the two largest being our small sample size and lack of external validation in a non-OAI cohort. We chose samples carefully in order to minimize group differences and reduce the possibility of batch effects, which we further reduced via *fsva* correction. Our findings are preliminary and should certainly be confirmed in a larger and more heterogeneous patient cohort. Unfortunately, our precise JSW measurement requirement^59^ and use of baseline peripheral blood DNA limits the possibility of direct comparison with other cohorts which are not as well characterized. These factors have led several previous studies to include only samples from the OAI^7,9,19,28–30,60–62^. Future studies will no doubt relax our inclusion criteria and expand the potential sources for additional samples to include larger sample sizes and additional cohorts.

A major concern with machine learning is generally the problem of overfitting. We reduced this possibility through extensive cross-validation and report the accuracy of models when tested on data unseen during model development. We included in our analysis data from both Illumina 450 k and 850 k chips, as the 450 k assays became unavailable from the manufacturer during this project. All data from 850k chips were subset to only those sites included on the 450k array and were batch corrected. We saw no evidence for differences in predictive capability with the two chip types (see above). We did see differences comparing M value-based to beta value-based models; however, this is not unsurprising given previous reports of variations in statistical measures of these values^24^ owing to differences in scedasticity. Finally, we are unable to draw substantial conclusions about underlying pathophysiological associations of altered PBMC DNA methylation patterns with OA given our mixed PBMC samples; this would be best investigated by future studies focusing on individual immune cell subsets. There is an emerging literature in the OA field which has demonstrated the importance of activated macrophages in disease development and progression^63–65^. We are unable to comment specifically on whether the nearly-statistically-significant increase in monocytes detected by computational estimation of PBMC composition which we found among progressors reflects this activated phenotype subset; however, future studies should certainly examine this possibility. It is important to highlight that the gene ontology analyses we performed herein were based on CpG sites selected following PBMC cell composition correction. Therefore, there is a possibility that additional, particularly macrophage- or monocyte-related, functional pathways and/or upstream regulators may have been missed by our analysis. These concerns do not, however, prevent us from performing predictive and prognostic biomarker analysis, as a clinically viable test would most easily include DNA from mixed PBMCs or whole blood.

Our data raise several questions which highlight potential avenues for future investigation. For example, it is unclear when epigenetic patterns change in the natural history of OA development and how stable they are over time; a longitudinal DNA methylation analysis in an incident OA cohort would be helpful in this regard. Our approach could also be used to evaluate PBMC epigenetic models for prediction of pain and combined pain and radiographic progression in larger OA cohorts. In this study, we did not seek to produce a consensus model by training on our entire dataset, as this would no doubt have produced significant model overfitting given our small sample size; rather, we sought to determine the feasibility and capability of PBMC methylation-based models for OA subtype discrimination more generally. Future studies on larger cohorts will no doubt have the ability to produce such a consensus model that would be of direct clinical relevance.

In summary, our pilot study provides the first analysis of peripheral blood epigenetic patterns as predictive models for knee OA radiographic progression. Our use of linear modeling applied to a large genome-wide DNA methylation dataset from well-matched case and control cohorts offer the first glimpse into the potential of future OA epigenetic classification. If confirmed, our approach could offer advantages over traditional biochemical biomarkers, including requiring a single baseline blood sample. Our results will certainly require additional validation in larger datasets but offer hope for the future development of easily accessible predictive biomarkers to personalize and improve the care of adults with knee OA.

## Methods

### Study design

This nested case-control study (116 total patients, 58 progressors, 58 nonprogressors) used data and biospecimens from the Osteoarthritis Initiative (OAI) database, which is available for public access at https://data-archive.nimh.nih.gov/oai/. All Osteoarthritis Initiative (OAI) participants provided written informed consent, and the study was carried out in accordance with the OAI data user agreement, approved by the Committee on Human Research of the Institutional Review Board (IRB) for the University of California, San Francisco (UCSF). The IRBs of the University of Oklahoma Health Sciences Center and Oklahoma Medical Research Foundation also reviewed and approved the project. Detailed case and control criteria are presented in Supplementary Document 1. Participants had baseline and yearly follow-up knee radiographs and PBMC DNA available. Kellgren-Lawrence Grade (KLG) and quantitative joint space width (JSW)^59^ were assessed by the central reading site using non-fluoroscopic fixed-flexion knee radiographs with a Synaflexer positioning device (Synaflexor, Synarc, Newark, CA). All participants had baseline KLG of 2–3 in at least one knee without a history of total knee joint replacement through 48-months. Participants with a tibial plateau rim distance of 6.5 mm at baseline, or with a change in the rim distance of >2.0 mm between baseline and follow-up were excluded due to unreliable radiographic positioning. Our case and control selection was limited by the availability of matched cases and controls, as below.

### Definition of radiographic progression, case and control group characteristics

Our definition of radiographic progression was similar to the case definitions of the OABC-FNIH^9^. Cases had radiographic progression in the medial tibiofemoral compartment by a longitudinal loss in the minimum JSW of at least 0.7 mm from baseline to 24-month follow-up in one index knee, with persistent narrowing in the same index knee at 48 months based on radiographs obtained from a non-fluoroscopic fixed flexion protocol (Synaflexor, Synarc, Newark, CA). In each case, the contralateral (non-index) knee had less progression than the index knee, or no progression over the follow-up period. Participants with a tibial plateau rim distance of 6.5 mm at baseline, or with a change in the rim distance of >2.0 mm between baseline and follow-up were excluded due to inappropriate and/or unreliable radiographic positioning. Non-progressors were defined as those with ≤0.5 mm of JSW loss from baseline to 48 months in either knee. These thresholds were set to coincide with previous OAI biomarker studies^9,60,62,66^, as defined in the Foundation for the National Institutes of Health biomarker consortium project (OABC-FNIH) and were set based on the distribution of 1-month change in minimum JSW in normal knees of OAI control participants and estimated to have ≤10% probability of change due to measurement error^8,67^.

Controls were frequency matched with cases by age category, sex, race, and BMI category as shown in Table 1 and Supplementary Table 1 and were also frequency matched with the larger OAI cohort to broaden the applicability of our findings. Group differences were calculated using a Student t-test and statistically analyzed using a 2-tailed P value. There were no differences among the two groups in any demographic category with the exception of mean baseline Western Ontario and McMaster (WOMAC) pain subscale (Table 1), although it should be noted that there was a trend towards fewer females included among cases (53% vs. 60%, p = 0.13), more NSAID use at baseline among cases (29% vs. 17%, p = 0.13), and an increased mean baseline K/L grade among cases (2.2 vs. 2.0, p = 0.09), although no difference in mean baseline JSW was noted in cases (3.9 vs. 4.0, p = 0.34). We also evaluated the presence of various comorbid conditions among data collected by the OAI, including history of heart attack, failure, stroke, pulmonary disease, diabetes, and cancer (Table 1). We adjusted our models for these variables as part of our analysis (see below).

### DNA methylation assays, PBMC composition assessment and adjustment

Five hundred nanograms of DNA was treated with sodium bisulfite (EZ DNA methylation kit, Zymo) and loaded onto Illumina Infinium HumanMethylation450k (n = 62 samples: 30 cases, 32 controls) or 850k (n = 54 samples: 28 cases, 26 controls) arrays. As no direct measures of PBMC cell type composition were available from the OAI, these data were estimated using the *estimateCellCounts* function of *minfi*. No statistically significant differences in estimated PBMC cell composition were found between groups (Table 1, Supplementary Table 2), although there was a trend towards increased monocytes among cases (7.4 ± 0.3% vs. 6.7 ± 0.3%, cases vs. controls mean ± SEM, p = 0.12). DNA methylation data were corrected for cell count variation using frozen surrogate variable analysis^22^ via the *sva* package (v. 3.28.0). This method has been previously shown to robustly correct for cell count variation and other batch effects in large-scale epigenomic studies^22,23,68^.

### Data preprocessing

Statistical analysis was performed using R (v. 3.5.0). Raw.IDAT files were processed using the *minfi* package (v. 1.26.2). Illumina 850k (EPIC) array data were subset to only those sites also included in the 450k array. Preprocessing and normalization were performed using the *preprocessFunnorm* function. All samples passed internal controls included on each chip and had detection p ≤ 0.01 for ≥97% of cytosine-guanosine dinucleotide (CpG) positions included on each array. Epigenome-wide association studies traditionally have used DNA methylation beta values, defined as the fraction methylation (0–1 scale) for a particular CpG site. Beta values, however, are characterized by high heteroscedasticity (most beta values fall within extreme high- and low-percent methylation levels), and questions have been raised regarding statistical validity of beta value analysis^24^. Therefore, we also analyzed M values (the log_2_ ratio of methylated:unmethylated probe intensities for a given CpG site), which are approximately homoscedastic. We excluded from analysis CpG probes located on sex chromosomes, probes with known single nucleotide polymorphisms (SNPs) with minor allele frequency of ≥5%, and probes not detected in all samples, leaving a final n = 435,118 CpG sites for analysis in each sample.

### Modeling

Elastic-net regularized generalized linear models were developed using the *glmnet* package (v. 2.0–16). As model overfitting is a frequently-occurring problem when developing classifiers on high-dimensional data, we implemented three strategies to reduce potential overfitting. First, we performed 7-fold internal cross-validation during development via the *cv*.*glmnet* function, utilizing the ‘one standard error rule’ when selecting lambda values^69^. Second, we tested models on lockbox data not used for training (Fig. 2). Finally, we repeated our model development and testing with 40 cycles of random splits of data into development and lockbox (validation) subsets. During each cycle of development, data were first randomly split into 60% development and 40% lockbox sets. Development data were then used to generate regularized cross-validated models and these models were tested on lockbox data and performance characteristics recorded. Model performance was assessed by the mean c-statistic (area under the receiver operator characteristic curve AUC-ROC), diagnostic odds ratio, accuracy, sensitivity, and specificity. The identity of DNA methylation sites (features) selected by *gmnet* for inclusion in each model, and the total number of sites required by each model, were recorded and compared. The performance of models by chip type (450k vs. 850k) was assessed by comparing accuracies using Fisher’s exact test of a 2 × 2 contingency table.

### Ontology analysis

Genes associated with CpG sites selected at least once during model development (n = 969) were analyzed using Ingenuity Pathway Analysis (IPA, Qiagen) v. 48207413 build 2019-06-16 using default settings.

## Supplementary information

Supplementary information

## Data Availability

The datasets generated during during the current study are available from the corresponding author on reasonable request.
