# Supplementary material for: A pilot study of peripheral blood DNA methylation models as predictors of knee osteoarthritis radiographic progression: data from the Osteoarthritis Initiative (OAI)
Source: Sci Rep. 2019 Nov 14;9:16880. doi: 10.1038/s41598-019-53298-9 (PMC6856188; doi:10.1038/s41598-019-53298-9)
Supplement: Supplementary file 1 — Supplementary information [file 41598_2019_53298_MOESM1_ESM.pdf]

Title:

A pilot study of peripheral blood DNA methylation models as predictors of knee osteoarthritis radiographic progression: data from the Osteoarthritis Initiative (OAI).

Authors:

Christopher Dunn[1,2]
Michael C. Nevitt[3]
John A. Lynch[3]
Matlock A. Jeffries[1,2]

1. University of Oklahoma Health Sciences Center, Department of Internal Medicine, Division of Rheumatology, Immunology, and Allergy, Oklahoma City, OK
2. Oklahoma Medical Research Foundation, Arthritis and Clinical Immunology Program, Oklahoma City, OK
3. University of California San Francisco, San Francisco, CA

Supplementary Information:

Supplementary Figure 1: Model performance by number of CpG sites included during development.

Supplementary Table 1: Extended case and control characteristics

Supplementary Table 2: CpG sites chosen during model development

Supplementary Document 1: Case and control eligibility and selection process

Supplementary Figure 1

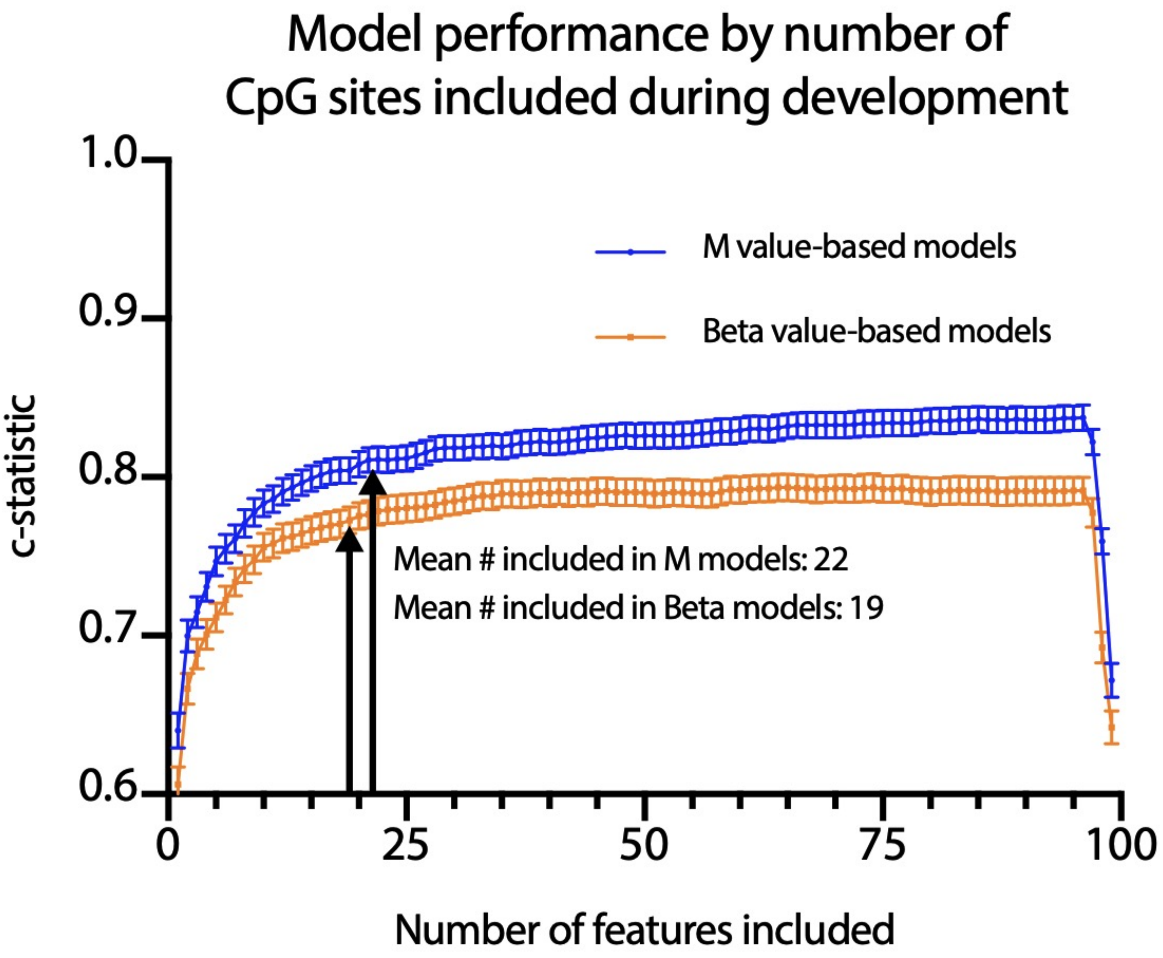

Supplementary Table 1: Extended case and control characteristics

Group	BMI Group	Age Group	BMI	Age	Ethnicity	Sex	Smoker (yes=1, no=0)	NSAID user (yes=1, no=0)	Mean bilateral baseline JSW (mm)	Mean bilateral baseline Western Ontario and McMasters Universities Osteoarthritis Index (WOMAC) pain score (0-20 scale)	Baseline K/L grade
Control	20.0-29.9	45-49	27.2	46	2: African American	male	0	1	3.74	2	2
Control	30.0+	60-69	30	66	1: Caucasian	female	1	0	4.39	3.5	2
Control	20.0-29.9	70-79	28.3	70	1: Caucasian	female	0	0	4.53	2.5	2
Control	20.0-29.9	60-69	26.8	69	1: Caucasian	female	0	0	4.67	0	2
Control	20.0-29.9	70-79	24.5	70	1: Caucasian	female	1	1	4.37	0	3
Control	20.0-29.9	45-49	25.9	48	1: Caucasian	male	0	0	4.57	2.5	2
Control	30.0+	60-69	33.2	66	1: Caucasian	female	1	0	4.41	0	2
Control	30.0+	60-69	33.4	60	1: Caucasian	female	0	0	3.46	1.5	2
Control	20.0-29.9	50-59	27.4	59	1: Caucasian	male	1	0	5.87	4.5	2
Case	20.0-29.9	60-69	29.5	65	2: African American	female	1	0	2.49	2.5	3
Control	20.0-29.9	50-59	29.7	59	1: Caucasian	female	0	0	4.25	1	3
Case	20.0-29.9	60-69	27.7	66	1: Caucasian	male	1	1	4.03	1.5	2
Control	30.0+	50-59	33.3	58	1: Caucasian	female	1	1	4.94	1	2
Case	30.0+	60-69	36.7	62	2: African American	female	0	0	3.21	8	3
Case	30.0+	50-59	34.2	50	2: African American	male	1	1	3.01	2.5	3
Case	30.0+	45-49	38.7	48	1: Caucasian	female	0	1	5.62	6.5	3
Control	30.0+	45-49	34.5	46	1: Caucasian	female	1	0	4.68	3	2
Case	30.0+	60-69	31.3	64	1: Caucasian	male	1	0	4.12	3	3
Control	30.0+	50-59	37.5	57	1: Caucasian	male	0	0	2.77	0	3
Control	30.0+	50-59	34	50	2: African American	female	1	1	4.17	9	2
Control	30.0+	45-49	30.4	46	1: Caucasian	male	0	0	4.05	0.5	3
Control	20.0-29.9	50-59	29.8	52	1: Caucasian	male	1	0	3.59	3.5	3
Case	20.0-29.9	45-49	27.6	46	1: Caucasian	female	1	0	5.26	3.5	2
Control	20.0-29.9	60-69	26.2	66	1: Caucasian	male	1	0	3.98	1	3
Case	30.0+	45-49	33.8	45	1: Caucasian	male	0	0	3.93	9.5	3
Control	20.0-29.9	50-59	27.4	53	1: Caucasian	male	0	0	3.95	1	2
Control	30.0+	45-49	32.3	48	1: Caucasian	female	0	1	5.45	4	2
Control	20.0-29.9	70-79	25.3	73	1: Caucasian	female	0	0	2.20	4	3
Case	20.0-29.9	50-59	28.2	54	1: Caucasian	male	0	1	4.40	5	3
Control	20.0-29.9	70-79	28.9	78	1: Caucasian	female	1	0	3.10	3	2
Case	30.0+	70-79	30.1	77	1: Caucasian	female	1	0	2.80	3	3
Control	30.0+	70-79	32.6	74	1: Caucasian	female	1	0	2.93	1	3
Case	30.0+	60-69	37.7	66	1: Caucasian	female	0	1	3.23	5.5	2
Control	20.0-29.9	50-59	28	52	1: Caucasian	male	0	1	3.61	1	2
Control	20.0-29.9	50-59	29	51	1: Caucasian	male	0	0	4.48	6.5	2
Case	20.0-29.9	50-59	29.1	52	1: Caucasian	male	1	0	4.03	7	2
Control	20.0-29.9	50-59	27.5	52	1: Caucasian	female	0	0	4.34	3	2
Control	30.0+	50-59	40.6	55	2: African American	male	1	0	3.78	0	3
Case	30.0+	45-49	34.3	48	1: Caucasian	female	0	0	4.09	10	3
Case	30.0+	50-59	31.1	57	2: African American	male	0	0	4.00	6	2
Control	30.0+	60-69	31.9	66	1: Caucasian	male	1	0	5.52	3	2
Control	30.0+	60-69	36.2	66	1: Caucasian	female	1	0	3.34	4	2
Case	20.0-29.9	50-59	28.8	59	1: Caucasian	male	0	1	3.10	3.5	3
Control	30.0+	50-59	30.1	54	1: Caucasian	male	0	0	5.01	4.5	2
Control	20.0-29.9	50-59	29.3	53	1: Caucasian	male	0	0	3.01	1	2
Control	30.0+	50-59	32.4	54	2: African American	male	1	0	**5.71**	12.5	2
Control	30.0+	50-59	30	51	2: African American	female	0	0	2.85	5.5	2
Case	20.0-29.9	50-59	29.2	59	1: Caucasian	male	1	0	1.87	8.5	3
Case	30.0+	50-59	34.5	53	1: Caucasian	male	0	1	4.23	4	3
Case	30.0+	60-69	32	67	1: Caucasian	male	0	0	4.66	2	2
Control	30.0+	50-59	30	51	1: Caucasian	female	1	0	4.01	0.5	2
Control	30.0+	60-69	30.6	62	1: Caucasian	male	0	0	4.60	1.5	3
Control	20.0-29.9	60-69	29.2	67	1: Caucasian	female	1	0	4.39	2	3
Case	30.0+	50-59	30.1	53	2: African American	female	1	0	5.40	9.5	2
Control	20.0-29.9	60-69	28.8	63	1: Caucasian	female	1	0	2.50	3.5	3
Case	30.0+	45-49	39.6	48	1: Caucasian	male	0	0	3.92	4.5	2
Case	30.0+	60-69	38.7	67	1: Caucasian	male	0	0	2.29	6	3
Case	20.0-29.9	60-69	26.5	66	1: Caucasian	male	1	0	2.70	7.5	3
Case	20.0-29.9	50-59	26.2	50	1: Caucasian	male	0	0	4.56	7.5	2
Control	20.0-29.9	60-69	28.4	63	1: Caucasian	female	0	1	3.92	0	2
Control	20.0-29.9	50-59	25.2	59	1: Caucasian	male	0	0	3.04	5	3
Control	20.0-29.9	70-79	28.8	71	1: Caucasian	male	0	0	2.83	0	3
Control	30.0+	50-59	31.5	51	1: Caucasian	male	1	1	4.78	4.5	2
Case	20.0-29.9	70-79	25.9	74	1: Caucasian	female	0	0	3.62	2	3
Case	20.0-29.9	50-59	26.5	52	1: Caucasian	male	0	0	5.45	4	2
Control	20.0-29.9	50-59	27.1	53	1: Caucasian	male	0	0	5.55	1	2
Control	30.0+	50-59	34.3	54	1: Caucasian	female	1	0	4.01	4	3
Case	20.0-29.9	50-59	22.4	53	1: Caucasian	male	1	1	3.17	2	3

Case	20.0-29.9	70-79	28.2	79 1: Caucasian	female	1	0	3.56	6.5	3
Case	20.0-29.9	60-69	28.8	67 1: Caucasian	male	1	0	6.97	4.5	2
Case	30.0+	60-69	33.3	62 1: Caucasian	female	1	1	4.10	4.5	3
Case	30.0+	50-59	32	54 2: African American	male	1	0	3.81	4	3
Case	20.0-29.9	50-59	29.7	50 1: Caucasian	female	0	0	4.01	3	2
Case	30.0+	60-69	36	62 1: Caucasian	male	1	1	2.01	3.5	3
Control	30.0+	60-69	30.4	65 1: Caucasian	male	0	0	5.27	0.5	2
Case	20.0-29.9	60-69	27.9	60 1: Caucasian	female	1	0	1.59	3	3
Control	20.0-29.9	60-69	28.6	65 1: Caucasian	male	1	0	4.80	8.5	3
Control	20.0-29.9	60-69	29.3	69 2: African American	female	0	1	5.27	5.5	2
Case	20.0-29.9	50-59	28.8	59 1: Caucasian	male	0	0	2.22	4.5	3
Case	20.0-29.9	70-79	26.3	78 1: Caucasian	male	0	0	1.49	5	3
Case	20.0-29.9	60-69	26	66 1: Caucasian	female	0	0	3.99	1	2
Control	30.0+	60-69	34.9	65 1: Caucasian	female	0	1	4.03	1.5	2
Case	30.0+	50-59	31.2	56 1: Caucasian	female	1	1	3.86	4.5	3
Case	20.0-29.9	60-69	27.6	60 1: Caucasian	male	1	0	4.33	4	2
Case	20.0-29.9	60-69	28	64 1: Caucasian	female	0	0	4.39	0.5	2
Case	20.0-29.9	60-69	25.2	69 1: Caucasian	female	0	0	2.78	2.5	3
Case	30.0+	50-59	31.8	53 1: Caucasian	female	1	1	4.02	2.5	2
Case	30.0+	60-69	31.3	67 1: Caucasian	male	1	0	5.16	13	2
Case	30.0+	50-59	37.9	50 1: Caucasian	male	1	0	4.63	2.5	3
Control	20.0-29.9	50-59	25.9	54 1: Caucasian	male	1	0	5.40	1.5	2
Control	30.0+	50-59	35.9	57 1: Caucasian	female	1	0	3.65	6	3
Case	30.0+	60-69	30.3	63 1: Caucasian	female	0	0	3.96	4	2
Case	20.0-29.9	50-59	25.6	58 1: Caucasian	female	0	0	2.65	4.5	3
Case	30.0+	50-59	33.6	59 1: Caucasian	male	1	1	2.99	0	3
Control	20.0-29.9	60-69	28.4	62 1: Caucasian	female	0	0	3.15	1.5	3
Case	20.0-29.9	50-59	29.1	53 1: Caucasian	male	0	0	5.25	1	2
Case	30.0+	50-59	30.1	53 1: Caucasian	female	0	0	5.12	2	2
Case	30.0+	50-59	30.6	53 1: Caucasian	female	0	0	2.95	5.5	3
Control	20.0-29.9	60-69	29	64 1: Caucasian	female	0	0	3.21	3	2
Case	20.0-29.9	45-49	29.5	47 2: African American	male	0	0	6.22	2	2
Case	30.0+	60-69	38.7	66 1: Caucasian	male	1	0	3.12	5	3
Control	30.0+	60-69	44.2	64 2: African American	female	0	0	3.20	12.5	2
Control	30.0+	45-49	41.1	49 1: Caucasian	female	0	0	4.62	11.15	2
Case	30.0+	60-69	30.4	66 1: Caucasian	female	1	0	5.14	2	2
Control	20.0-29.9	70-79	28.3	77 1: Caucasian	female	1	0	2.28	3	3
Case	20.0-29.9	50-59	25.3	58 1: Caucasian	male	0	0	5.38	0	2
Control	20.0-29.9	60-69	29.4	63 1: Caucasian	female	0	0	3.76	5	3
Control	30.0+	50-59	38.3	56 1: Caucasian	female	1	0	5.41	2	2
Control	30.0+	60-69	33.1	69 1: Caucasian	female	0	0	3.66	3.5	3
Case	20.0-29.9	70-79	26.6	70 1: Caucasian	female	0	1	2.69	8	3
Control	20.0-29.9	60-69	27.9	68 1: Caucasian	female	0	0	3.25	10	3
Case	20.0-29.9	70-79	23.9	70 1: Caucasian	female	0	0	3.38	6.5	2
Case	20.0-29.9	70-79	29.1	70 1: Caucasian	female	0	1	3.95	2.5	3
Control	30.0+	60-69	38.2	64 1: Caucasian	female	0	0	3.95	8	3
Case	20.0-29.9	60-69	29.2	69 1: Caucasian	male	1	1	4.44	3	3
Case	30.0+	50-59	35.1	50 1: Caucasian	female	1	1	4.01	0.5	2

Supplementary Table 2: CpG sites chosen during model development

TargetID	Beta count	M counts	GENOME_I	CHR	MAPINFO	UCSC_REFGENE_NAME	UCSC_REFGENE_ACCESSION	UCSC_REFGENE_GROU	UCSC_CPG_	RELATION_TO_UCSC_CPG_ISLAND
cg00002464		1	37	8	1.2E+08	NOV	NM_002514	TSS200	chr8:12042	Island
cg00027745		2	37	7	1452678				chr7:14524	Island
cg00089154	1		37	16	57352732					
cg00105102	2		37	1	1.56E+08	PAQR6;PAQR6	NM_024897;NM_198406	5'UTR;5'UTR	chr1:15621	S_Shore
cg00105102		2	37	1	1.56E+08	PAQR6;PAQR6	NM_024897;NM_198406	5'UTR;5'UTR	chr1:15621	S_Shore
cg00107890	1		37	15	71492746	THSD4	NM_024817	Body		
cg00125544	1		37	2	2.37E+08	AGAP1;AGAP1	NM_014914;NM_001037131	Body;Body	chr2:23686	N_Shore
cg00142933	1		37	2	1.28E+08	LIMS2;LIMS2;LIMS2;LIMS2	NM_001136037;NM_017980;NM_0011	Body;Body;Body;5'UTR	chr2:12842	N_Shore
cg00142933		8	37	2	1.28E+08	LIMS2;LIMS2;LIMS2;LIMS2	NM_001136037;NM_017980;NM_0011	Body;Body;Body;5'UTR	chr2:12842	N_Shore
cg00143249	5		37	7	1.57E+08	MNX1;MNX1	NM_001165255;NM_005515	Body;Body	chr7:15679	Island
cg00143249		1	37	7	1.57E+08	MNX1;MNX1	NM_001165255;NM_005515	Body;Body	chr7:15679	Island
cg00152637		1	37	6	28104690				chr6:28104	N_Shore
cg00180559	1		37	1	2.33E+08	KIAA1804	NM_032435	Body	chr1:23349	N_Shore
cg00192026	1		37	4	1.74E+08					
cg00278028	3		37	22	28196834	MN1;MN1	NM_002430;NM_002430	5'UTR;1stExon	chr22:2819	Island
cg00290605	1		37	22	32366707				chr22:3236	Island
cg00308618		1	37	2	1.53E+08	CACNB4;CACNB4;CACNB4;CACNB4	NM_000726;NM_001145798;NM_0010	Body;Body;TSS1500;Body		
cg00335286	2		37	18	13916355	MC2R	NM_000529	TSS1500		
cg00336071	1		37	15	63449943	RPS27L	NM_015920	TSS1500	chr15:6344	S_Shore
cg00355514	1		37	13	24914782				chr13:2491	Island
cg00386663		1	37	16	692659	FAM195A	NM_138418	Body	chr16:6915	Island
cg00388154	1		37	15	68498857	CALML4;CALML4	NM_001031733;NM_033429	TSS1500;TSS1500		
cg00401433	1		37	8	1.07E+08	ZFPM2	NM_012082	Body		
cg00409636		1	37	2	75720541	FAM176A;FAM176A	NM_001135032;NM_032181	Body;Body		
cg00466544	1		37	14	57046243	C14orf101	NM_017799	TSS1500	chr14:5704	N_Shore
cg00491335		1	37	2	2.25E+08				chr2:2247C	S_Shelf
cg00496805		1	37	19	57901154	ZNF548	NM_152909	TSS200	chr19:579C	Island
cg00497251	1		37	2	2.42E+08	RNPEPL1	NM_018226	5'UTR	chr2:2415C	S_Shore
cg00510552		2	37	16	4511987	NMRAL1	NM_020677	Body		
cg00551143		1	37	7	28998860	TRIL	NM_014817	TSS1500	chr7:28995	S_Shore
cg00562641	1		37	5	68789255	OCLN	NM_002538	5'UTR	chr5:68788	Island
cg00572243	1		37	8	28458806					
cg00587472		1	37	2	6952860					
cg00607154	1		37	5	6754116	POLS	NM_006999	Body	chr5:67551	N_Shore
cg00618507	1		37	7	1.02E+08	ORAI2;ORAI2	NM_032831;NM_001126340	3'UTR;3'UTR	chr7:10209	N_Shore
cg00704844	1		37	6	32821621	PSMB9;TAP1;PSMB9;TAP1	NM_002800;NM_000593;NM_148954;	TSS1500;5'UTR;TSS150	chr6:3282C	Island
cg00704844		1	37	6	32821621	PSMB9;TAP1;PSMB9;TAP1	NM_002800;NM_000593;NM_148954;	TSS1500;5'UTR;TSS150	chr6:3282C	Island
cg00708678		1	37	10	91294839	SLC16A12	NM_213606	5'UTR	chr10:9129	N_Shore
cg00712762	2		37	1	36351470	EIF2C1	NM_012199	Body	chr1:36348	S_Shore
cg00796164		1	37	19	57654968	ZIM3	NM_052882	5'UTR		
cg00819788	1		37	17	7308579	C17orf61	NM_152766	TSS1500	chr17:7307	Island
cg00859178	1		37	1	1.62E+08	UHMK1	NM_175866	TSS1500	chr1:16246	N_Shore
cg00878038		1	37	7	1.51E+08	WDR86	NM_198285	TSS1500	chr7:1511C	Island
cg00886571	1		37	14	1.05E+08				chr14:1052	Island
cg00891611	1		37	1	1.08E+08					
cg00943060		1	37	7	65211784				chr7:65215	N_Shelf
cg00989986		1	37	8	49746558				chr8:49747	N_Shore
cg01022859		1	37	17	60767314	MRC2	NM_006039	Body		
cg01029669	1		37	19	46999840	PNMAL2	NM_020709	TSS1500	chr19:4699	S_Shore

cg01062913		1	37	1	1.49E+08					
cg01090026		2	37	6	39692382	KIF6	NM_145027	Body	chr6:39692	N_Shore
cg01110552		1	37	10	1.04E+08	MGEA5;MGEA5	NM_001142434;NM_012215	TSS1500;TSS1500	chr10:1035	S_Shore
cg01120851	1		37	1	6657644	KLHL21	NM_014851	Body	chr1:66591	N_Shore
cg01125463	1		37	6	42946178	PEX6	NM_000287	1stExon	chr6:42946	N_Shore
cg01138671	1		37	6	32130686	PPT2;PPT2	NM_005155;NM_138717	Body;Body	chr6:32134	N_Shelf
cg01144965	1		37	8	1811760	ARHGEF10	NM_014629	Body		
cg01166564		1	37	2	26987492	C2orf18	NM_017877	Body	chr2:26987	Island
cg01198033	1		37	7	1.55E+08	CNPY1	NM_001103176	5'UTR	chr7:15530	Island
cg01232267	1		37	3	47398209				chr3:47397	Island
cg01263999		1	37	13	1.08E+08	FAM155A	NM_001080396	Body		
cg01309395		1	37	6	33091795	HLA-DPB2	NR_001435	Body		
cg01316857		2	37	19	48685724				chr19:4868	Island
cg01333080	1		37	3	1.84E+08	ECE2;ECE2;ECE2;ECE2	NM_001100120;NM_001037324;NM_0	Body;Body;Body;Body	chr3:18399	S_Shore
cg01338043	1		37	7	1E+08	AGFG2	NM_006076	TSS1500	chr7:10013	N_Shore
cg01392841	1		37	19	47016869				chr19:4701	Island
cg01427750		1	37	12	1.32E+08	GPR133	NM_198827	Body	chr12:1315	Island
cg01514015		1	37	1	2.36E+08	GPR137B	NM_003272	Body		
cg01515114	1		37	4	4139730					
cg01543583	1		37	14	59947673	C14orf149	NM_144581	Body	chr14:5995	N_Shelf
cg01548562	1		37	9	71836408	TJP2;TJP2;TJP2;TJP2;TJP2;TJP2	NM_001170414;NM_001170630;NM_0	Body;Body;Body;Body;	chr9:71835	Island
cg01565438		2	37	17	33776554	SLFN13	NM_144682	TSS1500	chr17:3377	Island
cg01591416	1		37	8	30601572	UBXN8	NM_005671	TSS200		
cg01620570	1		37	17	15165908	PMP22;PMP22	NM_153321;NM_000304	TSS200;5'UTR	chr17:1516	Island
cg01709473	1		37	6	12010960				chr6:12012	N_Shore
cg01709473		1	37	6	12010960				chr6:12012	N_Shore
cg01721429		1	37	16	47007486	DNAJA2;DNAJA2	NM_005880;NM_005880	5'UTR;1stExon	chr16:4700	Island
cg01771479	1		37	8	1.44E+08					
cg01867419		1	37	6	30421515				chr6:30418	S_Shore
cg01872216	1		37	14	54430382				chr14:5443	Island
cg01900413		1	37	11	1.28E+08	ETS1	NM_001143820	Body	chr11:1284	Island
cg01962086		1	37	3	1.96E+08	TNK2	NM_005781	TSS1500	chr3:19563	S_Shore
cg02055540		1	37	5	1.66E+08					
cg02071447	1		37	5	1.51E+08	GLRA1;GLRA1	NM_000171;NM_001146040	TSS200;TSS200	chr5:15130	Island
cg02148547		1	37	14	21457502	METT11D1;METT11D1	NM_001029991;NM_022734	TSS1500;TSS1500	chr14:2145	N_Shore
cg02192318		1	37	16	89500212	ANKRD11	NM_013275	5'UTR	chr16:8950	Island
cg02205746		1	37	16	2732724	KCTD5	NM_018992	1stExon	chr16:2732	Island
cg02215141	2		37	4	1.33E+08				chr4:13266	N_Shore
cg02215141		6	37	4	1.33E+08				chr4:13266	N_Shore
cg02216246	2		37	11	1.29E+08	FLI1;FLI1	NM_002017;NM_001167681	Body;5'UTR	chr11:1285	Island
cg02216246		1	37	11	1.29E+08	FLI1;FLI1	NM_002017;NM_001167681	Body;5'UTR	chr11:1285	Island
cg02241363	2		37	17	78450357	NPTX1;NPTX1	NM_002522;NM_002522	5'UTR;1stExon	chr17:7844	Island
cg02268171		2	37	13	24477610				chr13:2447	Island
cg02275394	1		37	13	80917432				chr13:8091	S_Shore
cg02275622	1		37	2	2.08E+08					
cg02286547		2	37	11	75920278				chr11:7591	Island
cg02301193	1		37	1	90267201					
cg02301193		1	37	1	90267201					
cg02309335	1		37	6	30227294	HLA-L	NR_027822	TSS200	chr6:30227	N_Shore
cg02339418	1		37	12	1.24E+08	C12orf65;C12orf65	NM_152269;NM_001143905	TSS200;TSS1500	chr12:1237	Island

cg02358630	1		37	10	71993457	PPA1	NM_021129	TSS1500	chr10:7199	Island
cg02368096	2		37	7	98248993	NPTX2	NM_002523	Body	chr7:98245	S_Shore
cg02398007	2		37	12	67616546					
cg02398007		1	37	12	67616546					
cg02444810		1	37	17	27055702	NEK8	NM_178170	TSS200	chr17:2705	Island
cg02448190	2		37	1	1.56E+08	RAB25	NM_020387	TSS1500		
cg02448190		1	37	1	1.56E+08	RAB25	NM_020387	TSS1500		
cg02491138		1	37	20	51589616	TSHZ2;TSHZ2	NM_173485;NM_173485	1stExon;5'UTR	chr20:5158	N_Shore
cg02503646	1		37	3	52444059	PHF7;PHF7;BAP1	NM_173341;NM_016483;NM_004656	TSS1500;TSS1500;TSS2	chr3:52443	Island
cg02504716		1	37	11	1.24E+08					
cg02529035		1	37	2	2.2E+08	ACCN4;ACCN4	NM_182847;NM_018674	TSS1500;TSS1500	chr2:22037	Island
cg02554361		1	37	16	11001757	CIITA	NM_000246	Body	chr16:1100	Island
cg02558537		1	37	11	1.07E+08	CWF19L2	NM_152434	TSS200	chr11:1073	Island
cg02574502	1		37	19	54486058	CACNG8	NM_031895	Body	chr19:5448	Island
cg02625590	1		37	19	41880276	TMEM91	NM_001098825	5'UTR	chr19:4188	N_Shelf
cg02633148		1	37	4	46391476	GABRA2;GABRA2	NM_001114175;NM_000807	TSS200;5'UTR	chr4:46391	N_Shore
cg02653364	1		37	16	58283706	CCDC113;CCDC113	NM_001142302;NM_014157	TSS200;TSS200	chr16:5828	Island
cg02704217		1	37	17	12568507	MYOCD;MYOCD	NM_153604;NM_001146312	TSS1500;TSS1500	chr17:1256	N_Shore
cg02721947	1		37	5	39719620				chr5:39721	N_Shore
cg02734521		1	37	6	1.08E+08					
cg02804100	1		37	19	720993	PALM;PALM	NM_002579;NM_001040134	Body;Body	chr19:7190	S_Shore
cg02811702	1		37	13	24901961				chr13:2490	N_Shore
cg02846648		1	37	12	1.29E+08	GLT1D1	NM_144669	Body		
cg02862362		1	37	6	1.32E+08	ARG1	NM_000045	Body		
cg02894027	1		37	2	17375162					
cg02904235		1	37	14	91527369	RPS6KA5;RPS6KA5	NM_182398;NM_004755	TSS1500;TSS1500	chr14:9152	Island
cg02947232		1	37	20	42937544	FITM2	NM_001080472	Body	chr20:4293	N_Shore
cg02962630	5		37	15	41222776	DLL4	NM_019074	Body	chr15:4121	Island
cg02962630		4	37	15	41222776	DLL4	NM_019074	Body	chr15:4121	Island
cg02991338	1		37	14	29236017	FOXG1	NM_005249	TSS1500	chr14:2923	N_Shore
cg03003722	2		37	6	38035488	ZFAND3	NM_021943	Body		
cg03004273		1	37	18	7568265	PTPRM;PTPRM	NM_002845;NM_001105244	Body;Body	chr18:7566	Island
cg03054162	1		37	2	70485230	PCYOX1;PCYOX1	NM_016297;NM_016297	1stExon;5'UTR	chr2:70485	N_Shore
cg03069383		1	37	2	18741691	RDH14	NM_020905	1stExon	chr2:18741	Island
cg03069736		1	37	17	72542117	CD300C;CD300C	NM_006678;NM_006678	1stExon;5'UTR		
cg03088705		1	37	10	1.12E+08	XPNPEP1;XPNPEP1;XPNPEP1	NR_030724;NM_001167604;NM_0203	Body;3'UTR;3'UTR		
cg03103023		1	37	1	25088406	CLIC4	NM_013943	Body		
cg03119288		1	37	19	10829265	DNM2;DNM2;DNM2;DNM2	NM_004945;NM_001005361;NM_001	Body;Body;Body;Body	chr19:1082	Island
cg03124231	1		37	4	1.74E+08	HMGB2;HMGB2;HMGB2;HMGB2	NM_001130689;NM_002129;NM_001	TSS1500;1stExon;TSS2	chr4:17425	Island
cg03212634	4		37	19	54292238					
cg03212634		9	37	19	54292238					
cg03243450	1		37	5	1800782	NDUFS6;MRPL36	NM_004553;NM_032479	TSS1500;TSS1500	chr5:17994	Island
cg03243450		5	37	5	1800782	NDUFS6;MRPL36	NM_004553;NM_032479	TSS1500;TSS1500	chr5:17994	Island
cg03249986	1		37	11	66383717	RBM14	NM_006328	TSS1500	chr11:6638	Island
cg03290040	1		37	22	24110355	CHCHD10	NM_213720	TSS1500	chr22:2411	Island
cg03290752	1		37	3	1.77E+08	TBL1XR1	NM_024665	TSS200	chr3:17691	N_Shore
cg03318904	1		37	22	39801522	MAP3K7IP1;MAP3K7IP1	NM_006116;NM_153497	Body;Body		
cg03331282	1		37	1	36056567	TFAP2E	NM_178548	Body		
cg03382797	1		37	9	1.36E+08	C9orf98;C9orf9	NM_152572;NM_018956	TSS1500;5'UTR	chr9:13575	S_Shore
cg03396103	1		37	12	1.17E+08				chr12:1169	Island

cg03396325	1		37	15	87876345					
cg03414134	2		37	3	1.87E+08	BCL6	NM_001706	5'UTR	chr3:1874	Island
cg03440588	3		37	1	47900320	FOXD2;MGC12982	NM_004474;NR_026878	TSS1500;TSS200	chr1:4789	Island
cg03440588		3	37	1	47900320	FOXD2;MGC12982	NM_004474;NR_026878	TSS1500;TSS200	chr1:4789	Island
cg03448396	1		37	3	38495949	LOC100128640;ACVR2B	NR_028389;NM_001106	Body;Body	chr3:3849	Island
cg03539051		1	37	19	56418660	NLRP13	NM_176810	Body		
cg03558399	2		37	3	67022555					
cg03558805		1	37	7	73184883	CLDN3	NM_001306	TSS1500	chr7:7318	Island
cg03586240	1		37	7	1966361	MAD1L1;MAD1L1;MAD1L1	NM_003550;NM_001013837;NM_001	Body;Body;Body	chr7:1967	N_Shore
cg03593908	1		37	13	60842299					
cg03597540		1	37	1	1.5E+08	HIST2H2BE;HIST2H2AC;HIST2H2BE	NM_003528;NM_003517;NM_003528	1stExon;TSS1500;3'UT	chr1:1498	N_Shore
cg03623968		1	37	3	1.29E+08	RAB7A	NM_004637	Body		
cg03632289	1		37	11	71301062					
cg03657549		2	37	7	1.28E+08	METTL2B;METTL2B	NM_018396;NM_018396	5'UTR;1stExon	chr7:1281	Island
cg03672997		1	37	10	7859623	TAF3	NM_031923	TSS1500	chr10:786	N_Shore
cg03677929	1		37	12	7342174	PEX5;PEX5;PEX5;PEX5;PEX5	NM_000319;NM_001131023;NM_001	TSS1500;TSS200;TSS20	chr12:734	Island
cg03677929		1	37	12	7342174	PEX5;PEX5;PEX5;PEX5;PEX5	NM_000319;NM_001131023;NM_001	TSS1500;TSS200;TSS20	chr12:734	Island
cg03687650	7		37	11	3144685	OSBPL5;OSBPL5;OSBPL5	NM_020896;NM_001144063;NM_145	Body;Body;Body	chr11:3141	S_Shelf
cg03687650		11	37	11	3144685	OSBPL5;OSBPL5;OSBPL5	NM_020896;NM_001144063;NM_145	Body;Body;Body	chr11:3141	S_Shelf
cg03703325		1	37	3	50606864	HEMK1	NM_016173	TSS200	chr3:5060	Island
cg03733229	2		37	21	46824701	COL18A1	NM_130445	TSS1500	chr21:4682	Island
cg03799405	2		37	4	1595726				chr4:15952	S_Shore
cg03822003		1	37	16	30569878	ZNF764	NM_033410	TSS1500	chr16:305	Island
cg03870777	10		37	12	53342755	KRT18;KRT18	NM_000224;NM_199187	TSS200;5'UTR	chr12:5334	N_Shore
cg03870777		6	37	12	53342755	KRT18;KRT18	NM_000224;NM_199187	TSS200;5'UTR	chr12:5334	N_Shore
cg03916104		1	37	1	6314748	GPR153	NM_207370	Body	chr1:63137	S_Shore
cg03928690	1		37	2	1.98E+08	MOBKL3;MOBKL3;MOBKL3	NM_199482;NM_001100819;NM_015	5'UTR;Body;Body	chr2:1983	Island
cg03937591	1		37	4	899553	GAK	NM_005255	Body	chr4:89977	N_Shore
cg04016326		1	37	12	14132940	GRIN2B;GRIN2B	NM_000834;NM_000834	1stExon;5'UTR	chr12:1413	N_Shore
cg04035728	1		37	8	27323467	CHRNA2	NM_000742	Body	chr8:2732	S_Shelf
cg04072156	1		37	3	1.49E+08	CPB1	NM_001871	TSS1500		
cg04083921	2		37	5	1.31E+08	RAPGEF6;RAPGEF6;RAPGEF6;RAPGEF6;RAPGE	NM_001164388;NM_001164386;NM_	Body;Body;Body;Body;Body;Body		
cg04101351		1	37	12	20522672	PDE3A	NM_000921	1stExon	chr12:2052	Island
cg04113418		1	37	19	49646093	PPFIA3	NM_003660	Body	chr19:4964	Island
cg04120546		1	37	8	1.26E+08	MTSS1	NM_014751	TSS200	chr8:12573	Island
cg04166812		1	37	14	61122051				chr14:6112	N_Shore
cg04171803		1	37	2	2.34E+08	GIGYF2;GIGYF2;GIGYF2;GIGYF2	NM_015575;NM_001103148;NM_001	TSS200;TSS200;TSS200	chr2:2335	N_Shore
cg04186622		1	37	1	1.49E+08				chr1:1485	Island
cg04199621		1	37	6	30955712	MUC21	NM_001010909	Body		
cg04215672	1		37	4	4108791				chr4:41087	Island
cg04223160		1	37	14	23755398	HOMEZ	NM_020834	TSS200	chr14:2375	Island
cg04254690		1	37	4	25031951	LGI2	NM_018176	Body	chr4:25032	N_Shore
cg04262428	2		37	11	70673256	SHANK2	NM_012309	Body	chr11:7067	S_Shore
cg04285141	2		37	16	57872427					
cg04285141		1	37	16	57872427					
cg04293489		1	37	6	49917881	DEFB133	NM_001166478	TSS1500		
cg04357082	1		37	16	980022	LMF1	NM_022773	Body	chr16:9797	Island
cg04357082		1	37	16	980022	LMF1	NM_022773	Body	chr16:9797	Island
cg04360049		1	37	1	1.58E+08	CD1D	NM_001766	5'UTR	chr1:1581	Island
cg04381865		1	37	16	15219673				chr16:1522	N_Shore

cg04406981		1	37	19	48453315	SNAR-C3	NR_024221	TSS1500		
cg04427003		4	37	5	63257499	HTR1A	NM_000524	1stExon	chr5:63256	Island
cg04441477		4	37	16	24267399	CACNG3;CACNG3	NM_006539;NM_006539	1stExon;5'UTR	chr16:2426	Island
cg04493430		1	37	16	1030388	SOX8	NM_014587	TSS1500	chr16:1029	Island
cg04498153	1		37	7	1.58E+08	PTPRN2;PTPRN2;PTPRN2	NM_002847;NM_130842;NM_130843	Body;Body;Body	chr7:15769	N_Shore
cg04498153		3	37	7	1.58E+08	PTPRN2;PTPRN2;PTPRN2	NM_002847;NM_130842;NM_130843	Body;Body;Body	chr7:15769	N_Shore
cg04529860		2	37	19	39900650				chr19:3990	Island
cg04544058		1	37	3	13609195	FBLN2;FBLN2;FBLN2	NM_001165035;NM_001004019;NM_(	TSS1500;5'UTR;5'UTR	chr3:13611	N_Shelf
cg04574459		1	37	21	47010768				chr21:4700	Island
cg04691540		1	37	1	1.79E+08	ABL2;ABL2;ABL2;ABL2;ABL2	NM_001136001;NM_001168238;NM_(	1stExon;1stExon;1stExo	chr1:17919	Island
cg04706600	2		37	5	1.79E+08	ADAMTS2;ADAMTS2	NM_021599;NM_014244	Body;Body		
cg04706600		4	37	5	1.79E+08	ADAMTS2;ADAMTS2	NM_021599;NM_014244	Body;Body		
cg04723723		1	37	1	67966270					
cg04729004		1	37	7	1.29E+08	KCP;KCP	NM_001135914;NM_199349	TSS200;TSS200	chr7:12855	S_Shore
cg04737185		1	37	11	1445041	BRSK2	NM_003957	Body		
cg04786330		1	37	12	27091356	C12orf11;FGFR1OP2;FGFR1OP2	NM_018164;NM_015633;NM_015633	TSS200;5'UTR;1stExon	chr12:2709	Island
cg04819714	1		37	11	1.01E+08	FLJ32810;TMEM133	NM_152432;NM_032021	3'UTR;TSS1500		
cg04821026	1		37	21	15068553				chr21:1506	N_Shore
cg04837170	1		37	16	1494876	CCDC154	NM_001143980	TSS1500	chr16:1495	N_Shore
cg04884908		2	37	2	72374840	CYP26B1	NM_019885	1stExon	chr2:72371	Island
cg04891086	1		37	12	5018513	KCNA1	NM_000217	TSS1500	chr12:5018	N_Shore
cg04899500	1		37	19	48965791	KCNJ14;KCNJ14	NM_170720;NM_013348	Body;Body	chr19:4896	Island
cg05025392	1		37	17	14738114					
cg05070971	2		37	21	43239518	PRDM15;PRDM15	NM_022115;NM_001040424	Body;Body	chr21:4324	N_Shore
cg05070971		1	37	21	43239518	PRDM15;PRDM15	NM_022115;NM_001040424	Body;Body	chr21:4324	N_Shore
cg05088794		1	37	18	33081137				chr18:3307	S_Shelf
cg05101846		1	37	17	72580962	C17orf77;CD300LD	NM_152460;NM_001115152	TSS200;Body		
cg05157470		1	37	6	28937310					
cg05229450		1	37	6	1.69E+08					
cg05240017	2		37	5	40756081	TTC33	NM_012382	TSS200	chr5:40755	Island
cg05240017		1	37	5	40756081	TTC33	NM_012382	TSS200	chr5:40755	Island
cg05242257	1		37	16	2588092	PDPK1;PDPK1;PDPK1;PDPK1	NM_031268;NM_002613;NM_031268;	5'UTR;1stExon;1stExon	chr16:2588	Island
cg05304037		1	37	19	50094202	PRR12;PRRG2	NM_020719;NM_000951	TSS1500;3'UTR	chr19:5009	Island
cg05377387		1	37	16	84328658	WFDC1	NM_021197	1stExon	chr16:8432	Island
cg05388057	1		37	1	78226545	USP33;USP33;USP33	NM_015017;NM_201626;NM_201624	TSS1500;TSS1500;TSS1	chr1:78224	S_Shore
cg05436007	1		37	6	1.47E+08					
cg05436007		1	37	6	1.47E+08					
cg05553502		1	37	5	92924091	NR2F1	NM_005654	Body	chr5:92923	Island
cg05587853	7		37	3	1.36E+08	MSL2;MSL2	NM_001145417;NM_018133	TSS1500;TSS200	chr3:13591	Island
cg05592114	1		37	12	7244104	C1R	NM_001733	Body		
cg05597349	1		37	10	1.03E+08	TLX1NB	NM_001085398	5'UTR	chr10:1028	Island
cg05634255		1	37	4	1.39E+08					
cg05668926	1		37	7	39609343	C7orf36	NM_020192	Body	chr7:39605	S_Shelf
cg05743054	2		37	3	1.58E+08	MLF1;MLF1;MLF1	NM_001130156;NM_022443;NM_0011	TSS200;TSS200;TSS200	chr3:15828	Island
cg05757007		1	37	6	1.01E+08	ASCC3	NM_006828	Body		
cg05835009		1	37	4	710272	PCGF3	NM_006315	5'UTR	chr4:71392	N_Shelf
cg05851240	1		37	18	72201663	CNDP1	NM_032649	TSS200		
cg05852760		1	37	7	23508224	IGF2BP3	NM_006547	Body	chr7:23508	Island
cg05854114	1		37	12	77719408				chr12:7771	Island
cg05907237		1	37	10	1.27E+08	CTBP2;CTBP2;CTBP2	NM_001083914;NM_001083914;NM_(	1stExon;5'UTR;TSS150(	chr10:1268	Island

cg05931119		1	37	6	1.69E+08					
cg05936555		2	37	6	30539508	ABCF1;ABCF1	NM_001090;NM_001025091	Body;Body	chr6:30538	S_Shore
cg05952475		1	37	6	1.64E+08	PACRG;PACRG;PACRG	NM_152410;NM_001080378;NM_001(	Body;Body;Body		
cg05963872		1	37	7	63652631				chr7:63652	Island
cg06016354		3	37	6	32163966	GPSM3;NOTCH4	NM_022107;NM_004557	TSS1500;Body	chr6:32163	Island
cg06039161		1	37	11	70672835	SHANK2	NM_012309	Body	chr11:7067	Island
cg06079468	1		37	10	81839181	LOC219347;LOC219347;LOC219347;C10orf57;I	NR_027430;NR_027431;NR_027428;NI	TSS1500;TSS1500;TSS1	chr10:8183	S_Shore
cg06081199		1	37	7	1.17E+08	CFTR;CFTR	NM_000492;NM_000492	1stExon;5'UTR		
cg06087019		1	37	3	27770182				chr3:27771	N_Shore
cg06090383		1	37	4	1.74E+08	SAP30	NM_003864	1stExon	chr4:17429	Island
cg06098215	2		37	10	51575702	NCOA4;NCOA4;NCOA4;NCOA4;NCOA4	NM_005437;NM_001145263;NM_0011	5'UTR;5'UTR;Body;TSS1	chr10:5157	S_Shelf
cg06153883	1		37	7	1.37E+08	CHRM2;CHRM2;CHRM2;CHRM2;CHRM2;CHRN	NM_001006631;NM_000739;NM_001(	5'UTR;5'UTR;5'UTR;5'UTR;5'UTR;5'UTR;5'UTR;5'UTR		
cg06166932	1		37	8	1.44E+08				chr8:14353	Island
cg06180519	2		37	10	32800689	CCDC7;CCDC7	NM_145023;NM_001026383	Body;Body		
cg06180519		3	37	10	32800689	CCDC7;CCDC7	NM_145023;NM_001026383	Body;Body		
cg06212297		1	37	16	48079666					
cg06254801	2		37	9	79379216	PCA3;PRUNE2	NR_015342;NM_015225	TSS200;Body		
cg06319785	1		37	2	75437207					
cg06326713		1	37	17	20745278				chr17:2074	N_Shore
cg06329022		5	37	17	26926511	SPAG5	NM_006461	TSS1500	chr17:2692	Island
cg06354519		1	37	1	27939417	FGR;FGR;FGR	NM_005248;NM_001042729;NM_001(	3'UTR;3'UTR;3'UTR	chr1:27941	N_Shelf
cg06409741	11		37	13	1.15E+08	RASA3	NM_007368	Body	chr13:1147	Island
cg06409741		11	37	13	1.15E+08	RASA3	NM_007368	Body	chr13:1147	Island
cg06444730	1		37	1	84544284	PRKACB;PRKACB	NM_002731;NM_207578	Body;Body	chr1:84543	S_Shore
cg06452419	1		37	17	55938940				chr17:5593	N_Shore
cg06456389	2		37	10	99052468	ARHGAP19	NM_032900	TSS200	chr10:9905	Island
cg06466917		1	37	5	17216259	LOC285696;BASP1	NR_027253;NM_006317	Body;TSS1500	chr5:17216	N_Shore
cg06483559	1		37	11	1.24E+08	OR6X1	NM_001005188	1stExon		
cg06502456	2		37	15	25304609	SNORD116-4	NR_003319	TSS200		
cg06502456		1	37	15	25304609	SNORD116-4	NR_003319	TSS200		
cg06515771		1	37	6	26614168				chr6:26614	Island
cg06521472	1		37	16	88990232	CBFA2T3;CBFA2T3	NM_175931;NM_005187	5'UTR;Body	chr16:8899	N_Shelf
cg06525193		1	37	2	2.38E+08					
cg06525491		5	37	11	71353991				chr11:7135	S_Shelf
cg06528306		1	37	8	53852661	NPBWR1	NM_005285	1stExon	chr8:53851	Island
cg06546406	2		37	1	39269756				chr1:39269	Island
cg06577604		1	37	3	1.84E+08	EPHB3	NM_004443	TSS200	chr3:18427	Island
cg06594186		1	37	7	12971260					
cg06638787	1		37	6	5997243				chr6:5997C	Island
cg06677013	1		37	6	84570388	CYB5R4	NM_016230	Body	chr6:84569	S_Shore
cg06681324	2		37	2	1.65E+08					
cg06681324		1	37	2	1.65E+08					
cg06731021		1	37	6	1.16E+08	COL10A1;NT5DC1	NM_000493;NM_152729	Body;Body		
cg06766273		2	37	11	62521983	ZBTB3	NM_024784	TSS1500	chr11:6252	S_Shore
cg06796869		1	37	1	2.28E+08	OBSCN;OBSCN	NM_052843;NM_001098623	Body;Body	chr1:22847	N_Shelf
cg06800849	1		37	16	89180587	ACSF3;ACSF3;ACSF3	NM_174917;NR_023316;NM_0011272	Body;Body;Body		
cg06816239	2		37	1	1.7E+08	SELL;SELL	NM_000655;NR_029467	Body;TSS1500		
cg06894723		1	37	13	1.1E+08					
cg06900229	1		37	17	48556928	RSAD1	NM_018346	Body	chr17:4855	S_Shore
cg06902609		2	37	7	985719	ADAP1	NM_006869	Body	chr7:98561	Island

cg06916315	1		37	5	1.74E+08				chr5:17417	Island
cg06996175	1		37	19	2546877	GNG7	NM_052847	5'UTR	chr19:2546	Island
cg07054668		1	37	3	52811637	ITIH1;ITIH1	NM_002215;NM_001166434	1stExon;TSS1500		
cg07061145	1		37	15	78556178	DNAJA4;DNAJA4	NM_018602;NM_001130182	TSS1500;TSS1500	chr15:7855	N_Shore
cg07089698		1	37	3	5161802				chr3:51638	N_Shelf
cg07102406	1		37	16	2294639	DCI	NM_001919	Body		
cg07102406		3	37	16	2294639	DCI	NM_001919	Body		
cg07115206		1	37	16	6425686	A2BP1;A2BP1	NM_001142333;NM_018723	5'UTR;5'UTR		
cg07119830	1		37	10	1.04E+08	TRIM8	NM_030912	Body		
cg07139514	1		37	2	10665727				chr2:10666	N_Shore
cg07145255		1	37	5	59995354	DEPDC1B;DEPDC1B	NM_018369;NM_001145208	Body;Body	chr5:59995	N_Shore
cg07163173		1	37	2	67624373	ETAA1	NM_019002	TSS200	chr2:67624	Island
cg07165115		1	37	2	82756475					
cg07165756		1	37	11	66311422	ZDHHC24	NM_207340	Body	chr11:6631	N_Shore
cg07170752	1		37	1	2.25E+08	DNAH14	NM_001373	Body		
cg07184658	2		37	9	96722329				chr9:96720	Island
cg07190543		1	37	7	8261154	ICA1;ICA1;ICA1	NM_004968;NM_001136020;NM_0223	Body;Body;Body		
cg07216337		1	37	8	75233610	JPH1	NM_020647	TSS200	chr8:75232	Island
cg07258847	2		37	6	66708570					
cg07258847		6	37	6	66708570					
cg07266964	1		37	2	1.36E+08					
cg07276861		1	37	4	76862355	NAAA;NAAA	NM_014435;NM_001042402	TSS200;TSS200	chr4:76861	Island
cg07288557		3	37	12	47226301				chr12:4722	S_Shore
cg07304175	1		37	3	1.81E+08	SOX2OT	NR_004053	Body	chr3:18141	S_Shore
cg07321237		1	37	7	82754608	PCLO;PCLO	NM_014510;NM_033026	Body;Body		
cg07331204		1	37	6	74998385					
cg07367469		1	37	5	1.16E+08					
cg07379140	5		37	1	95538546	ALG14	NM_144988	TSS200	chr1:95538	Island
cg07379140		1	37	1	95538546	ALG14	NM_144988	TSS200	chr1:95538	Island
cg07476643		1	37	10	1.03E+08				chr10:1027	N_Shore
cg07483064	1		37	1	8938751	ENO1;ENO1	NM_001428;NM_001428	1stExon;5'UTR	chr1:89380	Island
cg07487925		1	37	17	25620950	WSB1;WSB1	NM_015626;NM_134265	TSS200;TSS200	chr17:2562	N_Shore
cg07493834		1	37	2	22114272					
cg07499372		1	37	19	57631232	USP29	NM_020903	TSS1500	chr19:5763	S_Shore
cg07517948	1		37	15	67801494					
cg07549590		1	37	16	15018862				chr16:1501	Island
cg07554478	1		37	3	1.13E+08	ATP6V1A	NM_001690	5'UTR		
cg07562918	2		37	9	21994435	CDKN2A;CDKN2BAS	NM_058195;NR_003529	1stExon;TSS1500	chr9:21994	Island
cg07576142	1		37	13	93879769	GPC6	NM_005708	1stExon	chr13:9387	Island
cg07581226		2	37	2	2939627					
cg07588141	1		37	3	1.06E+08	CBLB	NM_170662	TSS1500	chr3:10558	Island
cg07596845		1	37	6	82957841	IBTK	NM_015525	TSS1500	chr6:82957	Island
cg07602984	1		37	1	36790927	FAM176B	NM_018166	TSS1500	chr1:36786	S_Shore
cg07605410		1	37	1	16828163				chr1:16825	S_Shore
cg07633317		1	37	16	49732623	ZNF423	NM_015069	Body	chr16:4973	Island
cg07695475	1		37	15	1.02E+08	OR4F15	NM_001001674	1stExon		
cg07741793	1		37	17	26989030	SDF2;SUPT6H	NM_006923;NM_003170	TSS200;TSS1500	chr17:2698	Island
cg07743117		2	37	5	77146278				chr5:77146	N_Shore
cg07749943		1	37	4	3468188	DOK7;DOK7	NM_173660;NM_001164673	Body;Body	chr4:34647	S_Shelf
cg07772660	1		37	1	17085863	MST1P9	NR_002729	Body	chr1:17085	Island

cg07772660		9	37	1	17085863	MST1P9	NR_002729	Body	chr1:17085	Island
cg07781162	1		37	17	74533951	CYGB	NM_134268	TSS200	chr17:7453	Island
cg07812819	1		37	7	89975908	GTPBP10;GTPBP10	NM_001042717;NM_033107	TSS200;TSS200	chr7:89975	Island
cg07813249	1		37	4	46392253	GABRA2;GABRA2	NM_000807;NM_001114175	TSS200;TSS1500	chr4:46391	Island
cg07813747		1	37	16	21514953	LOC100271836	NR_027155	TSS1500	chr16:2151	S_Shore
cg07817024		1	37	1	1.2E+08				chr1:11953	N_Shore
cg07835814	1		37	20	32077669	CBFA2T2	NM_001032999	TSS1500	chr20:3207	Island
cg07864467	1		37	10	1.34E+08					
cg07883117	1		37	7	1.01E+08	EMID2	NM_133457	TSS1500	chr7:1010C	N_Shore
cg07908805		2	37	19	427022	SHC2	NM_012435	Body	chr19:4267	Island
cg07919744	1		37	15	52873609	KIAA1370	NM_019600	3'UTR		
cg07962315		1	37	9	77704473	OSTF1;C9orf95;C9orf95;C9orf95	NM_012383;NR_023352;NM_0011276	Body;TSS1500;TSS1500	chr9:77702	S_Shore
cg07966388		1	37	15	93363564					
cg08028004		1	37	2	1.09E+08	RANBP2;RANBP2	NM_006267;NM_006267	1stExon;5'UTR	chr2:10933	Island
cg08035555		1	37	19	3558417	C19orf28;C19orf28;C19orf28	NM_021731;NM_001042680;NM_1749	TSS1500;TSS1500;TSS1	chr19:3556	S_Shore
cg08076266	1		37	2	1.2E+08	TMEM37	NM_183240	Body	chr2:12019	Island
cg08237220	1		37	3	1.56E+08					
cg08242475		1	37	7	1327791				chr7:13285	N_Shore
cg08269037		1	37	1	11751616	MAD2L2;C1orf187;MAD2L2	NM_001127325;NM_198545;NM_0011	5'UTR;TSS200;1stExon	chr1:1175C	Island
cg08318726	1		37	12	1.15E+08	TBX5;TBX5;TBX5	NM_181486;NM_000192;NM_080717	TSS1500;5'UTR;5'UTR	chr12:1148	N_Shore
cg08356503	1		37	16	88105836	BANP;BANP	NM_017869;NM_079837	Body;Body	chr16:8810	Island
cg08376824	1		37	20	5987738	CRLS1;CRLS1	NM_019095;NM_001127458	Body;TSS200	chr20:5986	S_Shore
cg08447792	1		37	4	1.11E+08	CCDC109B	NM_017918	Body		
cg08453096		1	37	2	44065893	ABCG5;ABCG8;ABCG5	NM_022436;NM_022437;NM_022436	5'UTR;TSS1500;1stExon		
cg08476757		1	37	3	1.08E+08	IFT57	NM_018010	Body	chr3:10794	Island
cg08514765		2	37	16	58058888	MMP15	NM_002428	TSS1500	chr16:5805	Island
cg08521684		1	37	3	1.95E+08	MUC4;MUC4;MUC4	NM_018406;NM_138297;NM_004532	Body;Body;Body	chr3:19548	Island
cg08561442	1		37	16	49891857				chr16:4988	Island
cg08592761		1	37	15	71146185	LARP6;LARP6	NM_197958;NM_018357	Body;Body	chr15:7114	Island
cg08623277		1	37	2	2.06E+08	PARD3B;PARD3B;PARD3B	NM_152526;NM_057177;NM_205863	Body;Body;Body		
cg08643994		1	37	4	1.42E+08					
cg08658621	1		37	10	1.34E+08	DPYSL4	NM_006426	TSS1500	chr10:1339	Island
cg08671647	1		37	4	30724564	PCDH7;PCDH7;PCDH7	NM_032456;NM_032457;NM_002589	1stExon;Body;1stExon	chr4:30721	Island
cg08671647		2	37	4	30724564	PCDH7;PCDH7;PCDH7	NM_032456;NM_032457;NM_002589	1stExon;Body;1stExon	chr4:30721	Island
cg08727867	1		37	21	15588349	RBM11	NM_144770	TSS200	chr21:1558	N_Shore
cg08728848	6		37	17	30437958					
cg08728848		3	37	17	30437958					
cg08751971	1		37	7	1.29E+08	IRF5;IRF5;IRF5;IRF5;IRF5;IRF5;IRF5	NM_032643;NM_001098629;NM_0010	Body;Body;Body;Body;Body;Body;Body		
cg08753297	2		37	6	56716612	DST;DST;DST;DST	NM_001144771;NM_001144770;NM_(	TSS200;5'UTR;1stExon;Body		
cg08782158	4		37	2	927755					
cg08813539		1	37	10	29011452				chr10:2901	Island
cg08852245	1		37	4	1.75E+08				chr4:17513	S_Shelf
cg08864035		3	37	1	1.14E+08	HIPK1;HIPK1	NM_198268;NM_152696	TSS1500;TSS1500	chr1:11447	N_Shore
cg08866633	1		37	13	1.14E+08	LAMP1	NM_005561	Body		
cg08891883	1		37	4	8347266				chr4:83474	N_Shore
cg08905496	3		37	1	1.65E+08	RXRG;RXRG;RXRG;RXRG	NM_001009598;NM_006917;NM_0069	5'UTR;5'UTR;1stExon;1stExon		
cg08943292	1		37	4	40337370	CHRNA9	NM_017581	TSS200		
cg08983429	1		37	16	3577455	CLUAP1;CLUAP1	NM_015041;NM_024793	Body;Body		
cg08992729		1	37	6	32809425	PSMB8;PSMB8	NM_004159;NM_148919	Body;Body	chr6:32811	N_Shelf
cg09032973	1		37	5	1.4E+08	PCDHA1;PCDHA1;PCDHA1	NM_031410;NM_031411;NM_018900	1stExon;1stExon;1stExo	chr5:14016	Island

cg09119967	1		37	20	9494838	C20orf103	NM_012261	TSS1500	chr20:9495	N_Shore
cg09214993	1		37	15	64673618	KIAA0101;KIAA0101;KIAA0101;KIAA0101	NM_001029989;NM_014736;NM_0010	1stExon;1stExon;5'UTR;5'UTR		
cg09222892		1	37	1	25734099	RHCE;RHCE;RHCE;RHCE	NM_138616;NM_138617;NM_138618;	Body;Body;Body;Body		
cg09272909	1		37	9	71736999	TJP2	NM_001170414	5'UTR	chr9:71736	Island
cg09292409		2	37	16	15461083					
cg09334915	1		37	1	2.39E+08					
cg09343402	1		37	19	24216269					
cg09365557		1	37	17	59477564	TBX2	NM_005994	1stExon	chr17:5947	Island
cg09413529	1		37	12	1.15E+08	TBX3;TBX3	NM_005996;NM_016569	Body;Body	chr12:1151	Island
cg09418673	3		37	11	76372128	LRRC32;LRRC32	NM_001128922;NM_005512	Body;Body		
cg09418673		4	37	11	76372128	LRRC32;LRRC32	NM_001128922;NM_005512	Body;Body		
cg09463211	1		37	15	99093248				chr15:9909	S_Shore
cg09499421	1		37	16	31020735	STX1B	NM_052874	Body		
cg09507411	1		37	5	82395650	XRCC4;XRCC4;XRCC4	NM_022550;NM_003401;NM_022406	5'UTR;5'UTR;5'UTR		
cg09600829		2	37	9	96216052	FAM120A;FAM120AOS	NM_014612;NM_198841	Body;TSS200	chr9:96213	S_Shore
cg09653641	1		37	7	92455499	CDK6;CDK6	NM_001259;NM_001145306	Body;Body		
cg09672383	2		37	2	25390540	POMC;POMC	NM_000939;NM_001035256	5'UTR;5'UTR	chr2:25391	N_Shore
cg09672383		1	37	2	25390540	POMC;POMC	NM_000939;NM_001035256	5'UTR;5'UTR	chr2:25391	N_Shore
cg09717927		1	37	13	1.13E+08				chr13:1126	N_Shore
cg09788029	3		37	12	1.03E+08					
cg09814034		1	37	20	44876346	CDH22	NM_021248	Body	chr20:4487	S_Shore
cg09822136	1		37	5	14144123	TRIO	NM_007118	Body	chr5:14143	Island
cg09952395	1		37	11	63258712	HRASLS5;HRASLS5;HRASLS5	NM_001146728;NM_001146729;NM_0	TSS200;TSS200;TSS200	chr11:6325	Island
cg10006235		2	37	11	49226370	FOLH1;FOLH1	NM_004476;NM_001014986	Body;Body	chr11:4922	N_Shelf
cg10045446	1		37	7	32530734	LSM5;LSM5;LSM5;LSM5	NM_001139499;NM_012322;NR_0244	5'UTR;TSS1500;TSS150	chr7:32529	S_Shore
cg10045446		2	37	7	32530734	LSM5;LSM5;LSM5;LSM5	NM_001139499;NM_012322;NR_0244	5'UTR;TSS1500;TSS150	chr7:32529	S_Shore
cg10077985	1		37	10	833127					
cg10091458	3		37	9	1.17E+08	KIF12	NM_138424	Body	chr9:1168	N_Shore
cg10128416		1	37	1	75198403	TYW3;CRYZ;CRYZ;TYW3;TYW3;CRYZ;CRYZ	NM_001162916;NM_001130042;NM_0	TSS1500;5'UTR;5'UTR;TSS1500;TSS1500;5'UTR;5'UTR		
cg10159215		1	37	1	99470560	LPPR5;LPPR5	NM_001010861;NM_001037317	TSS200;TSS200	chr1:99469	Island
cg10174031		1	37	16	31408898	ITGAD	NM_005353	Body		
cg10202874		1	37	16	32165225	HERC2P4	NR_002827	TSS1500		
cg10206440	4		37	1	16353594	CLCNKA;CLCNKA	NM_001042704;NM_004070	Body;Body		
cg10206440		1	37	1	16353594	CLCNKA;CLCNKA	NM_001042704;NM_004070	Body;Body		
cg10211414	1		37	1	8875053	RERE;RERE	NM_012102;NM_001042681	5'UTR;5'UTR	chr1:8877	N_Shore
cg10211414		1	37	1	8875053	RERE;RERE	NM_012102;NM_001042681	5'UTR;5'UTR	chr1:8877	N_Shore
cg10261325	1		37	10	29874759	SVIL;SVIL	NM_021738;NM_003174	5'UTR;5'UTR		
cg10261325		1	37	10	29874759	SVIL;SVIL	NM_021738;NM_003174	5'UTR;5'UTR		
cg10306485	7		37	13	1.15E+08	RASA3	NM_007368	Body	chr13:1148	S_Shelf
cg10306485		3	37	13	1.15E+08	RASA3	NM_007368	Body	chr13:1148	S_Shelf
cg10368533		1	37	12	22778127	ETNK1;ETNK1	NM_001039481;NM_018638	1stExon;1stExon	chr12:2277	Island
cg10368935	1		37	10	18240316	SLC39A12;SLC39A12	NM_001145195;NM_152725	TSS1500;TSS1500		
cg10405239		1	37	17	26733360	SLC46A1	NM_080669	TSS200	chr17:2673	Island
cg10423227	1		37	2	64752200	AFTPH;AFTPH;AFTPH	NM_203437;NM_001002243;NM_0176	5'UTR;5'UTR;5'UTR	chr2:64751	S_Shore
cg10490795	1		37	5	1.5E+08	SYNPO	NM_007286	Body	chr5:15003	N_Shore
cg10514235		1	37	4	15003844				chr4:15003	Island
cg10579818	1		37	19	34117242	CHST8;CHST8	NM_001127895;NM_001127896	5'UTR;5'UTR	chr19:3411	S_Shelf
cg10596590		1	37	6	51814836	PKHD1;PKHD1	NM_138694;NM_170724	Body;Body		
cg10601972		1	37	3	12851576	CAND2;CAND2	NM_001162499;NM_012298	Body;Body	chr3:12851	Island
cg10607939		1	37	7	1.12E+08	IFRD1;IFRD1	NM_001550;NM_001007245	Body;Body	chr7:11209	Island

cg10609068	4		37	12	45609265	ANO6;ANO6;PLEKHA9;ANO6	NM_001142680;NM_001142679;NM_(	TSS1500;TSS1500;5'UT	chr12:4560	N_Shore
cg10609068		1	37	12	45609265	ANO6;ANO6;PLEKHA9;ANO6	NM_001142680;NM_001142679;NM_(	TSS1500;TSS1500;5'UT	chr12:4560	N_Shore
cg10674754	4		37	4	8456019	C4orf23	NM_152544	TSS200		
cg10674754		3	37	4	8456019	C4orf23	NM_152544	TSS200		
cg10688514	1		37	10	48354852	ZNF488	NM_153034	TSS1500	chr10:4835	Island
cg10703619	1		37	7	1.55E+08					
cg10711996	1		37	10	32635917	EPC1;EPC1	NM_025209;NM_025209	5'UTR;1stExon	chr10:3263	Island
cg10722444		1	37	13	1.13E+08				chr13:1126	Island
cg10776643	1		37	14	71527576	PCNX	NM_014982	Body		
cg10842913	1		37	12	1.26E+08					
cg10843276	1		37	4	726697	PCGF3	NM_006315	5'UTR	chr4:72433	S_Shore
cg10895973		1	37	17	14212636	HS3ST3B1	NM_006041	Body	chr17:1421	Island
cg10902101	1		37	22	48899955	FAM19A5	NM_001082967	Body	chr22:4889	S_Shelf
cg10909154		1	37	16	2723619				chr16:2722	Island
cg10932815	1		37	12	68294240					
cg10966582	1		37	1	17085860	MST1P9	NR_002729	Body	chr1:17085	Island
cg10966582		5	37	1	17085860	MST1P9	NR_002729	Body	chr1:17085	Island
cg10988062		1	37	7	64023434	ZNF680;ZNF680;ZNF680;ZNF680	NM_001130022;NM_178558;NM_1785	1stExon;5'UTR;1stExon;5'UTR		
cg11005250		3	37	12	1.08E+08				chr12:1082	Island
cg11084629		1	37	1	55230770	PARS2	NM_152268	TSS1500	chr1:55229	S_Shore
cg11104313	1		37	3	1.12E+08	TAGLN3;TAGLN3;TAGLN3	NM_013259;NM_001008272;NM_001(	TSS200;TSS200;TSS1500		
cg11159141		1	37	3	11529333	ATG7;ATG7;ATG7	NM_001136031;NM_001144912;NM_(	Body;Body;Body	chr3:11529	N_Shore
cg11199172	1		37	17	46707338				chr17:4671	N_Shelf
cg11203397	1		37	13	46870454					
cg11222065	1		37	6	33240468	VPS52;RPS18	NM_022553;NM_022551	TSS1500;Body	chr6:33239	S_Shore
cg11273848		1	37	10	1.31E+08				chr10:1305	N_Shore
cg11303425		1	37	12	6798826	ZNF384;ZNF384;ZNF384;ZNF384;ZNF384;ZNF3	NM_001039916;NM_001039920;NM_(	TSS1500;TSS200;TSS20	chr12:6798	Island
cg11322849		1	37	11	2182783	INS;INS-IGF2;INS-IGF2	NM_000207;NR_003512;NM_0010423	TSS1500;TSS1500;TSS1500		
cg11336643		1	37	12	1.24E+08	CCDC92	NM_025140	5'UTR		
cg11350208		1	37	2	1.33E+08					
cg11386711	1		37	3	1.87E+08	ST6GAL1;ST6GAL1	NM_173216;NM_173217	5'UTR;5'UTR	chr3:18664	S_Shelf
cg11461030	1		37	1	46932239				chr1:46932	Island
cg11461302	1		37	22	38507451	BAIAP2L2	NM_025045	TSS1500		
cg11498652	2		37	5	40834691	RPL37	NM_000997	Body	chr5:40835	N_Shore
cg11542394	2		37	15	1.01E+08	ALDH1A3	NM_000693	TSS1500	chr15:1014	N_Shore
cg11551464		3	37	8	49595738					
cg11553116		1	37	6	2871823				chr6:28756	N_Shelf
cg11575929		1	37	6	28414894					
cg11677722		1	37	19	39881393	MED29;PAF1	NM_017592;NM_019088	TSS1500;Body	chr19:3988	Island
cg11723795	1		37	14	91975931	SMEK1	NM_032560	Body	chr14:9197	Island
cg11746846	3		37	18	3067337	MYOM1;MYOM1	NM_019856;NM_003803	Body;Body	chr18:3067	Island
cg11746846		1	37	18	3067337	MYOM1;MYOM1	NM_019856;NM_003803	Body;Body	chr18:3067	Island
cg11750736	1		37	17	10634140	TMEM220	NM_001004313	TSS1500	chr17:1063	S_Shore
cg11781564	1		37	5	73016764	RGNEF	NM_001080479	Body		
cg11801727		1	37	1	2.29E+08				chr1:22865	Island
cg11832722	1		37	18	28623060	DSC3;DSC3	NM_001941;NM_024423	TSS1500;TSS1500	chr18:2862	Island
cg11865413	15		37	10	72969054				chr10:7297	N_Shelf
cg11865413		9	37	10	72969054				chr10:7297	N_Shelf
cg11958128	1		37	12	3226445	TSPAN9;TSPAN9	NM_001168320;NM_006675	5'UTR;5'UTR		
cg12000587	1		37	17	30186630	C17orf79	NM_018405	TSS1500	chr17:3018	S_Shore

cg12014145		1	37	16	30346812	LOC595101	NR_002453	TSS200	chr16:3034	Island
cg12015279	1		37	18	55318716	ATP8B1	NM_005603	Body	chr18:5531	S_Shelf
cg12041387		2	37	7	96650171	DLX5	NM_005221	Body	chr7:9665C	N_Shore
cg12068833	1		37	4	1.33E+08				chr4:1328S	N_Shore
cg12068833		1	37	4	1.33E+08				chr4:1328S	N_Shore
cg12071328	1		37	11	20690930	NELL1;NELL1	NM_201551;NM_006157	TSS200;TSS200	chr11:206S	Island
cg12102432	1		37	11	1.19E+08					
cg12226306	1		37	3	1.05E+08	ALCAM	NM_001627	Body	chr3:10508	N_Shore
cg12226306		1	37	3	1.05E+08	ALCAM	NM_001627	Body	chr3:10508	N_Shore
cg12243858		1	37	7	1.49E+08	SSPO	NM_198455	1stExon	chr7:1494€	S_Shelf
cg12285737		1	37	19	49931759				chr19:4993	Island
cg12329460		1	37	10	1.06E+08	C10orf79	NM_025145	TSS200	chr10:105S	Island
cg12378753	1		37	10	1.17E+08				chr10:1165	N_Shore
cg12400434		1	37	5	1491377	LPCAT1	NM_024830	Body	chr5:14897	S_Shore
cg12458866	1		37	1	9353610	SPSB1	NM_025106	5'UTR		
cg12466737	2		37	18	35146589	BRUNOL4;BRUNOL4;BRUNOL4;BRUNOL4	NM_001025089;NM_001025087;NM_(	TSS1500;TSS1500;TSS1	chr18:3514	Island
cg12467960	2		37	11	68201194	LRP5	NM_002335	Body	chr11:682C	Island
cg12473849	1		37	3	1.37E+08					
cg12489353		1	37	19	48231499	EHD2	NM_014601	Body	chr19:4822	S_Shore
cg12494529	1		37	10	1082432	C10orf110;C10orf110;C10orf110;C10orf110	NR_024629;NR_027708;NR_027709;NF	Body;Body;Body;Body		
cg12512039		1	37	7	65958832				chr7:6595E	Island
cg12522342	2		37	17	64831573				chr17:6483	Island
cg12532563		1	37	4	83956223	COPS4	NM_016129	TSS200		
cg12568669	1		37	8	11666485	FDFT1	NM_004462	Body	chr8:11665	S_Shore
cg12583367		1	37	4	4412229	D4S234E;D4S234E	NM_014392;NM_001040101	Body;Body		
cg12624829	2		37	10	1.06E+08	CCDC147	NM_001008723	TSS1500		
cg12791476		1	37	7	44059249	POLR2J4	NR_003655	TSS1500	chr7:4405E	S_Shore
cg12797594	1		37	22	36236395	RBM9;RBM9;RBM9;RBM9;RBM9;RBM9;RBM9;	NM_014309;NM_001082578;NM_001(	5'UTR;Body;Body;5'UTR;5'UTR;1stExon;1stExon;1stExon;5'UTR;1stExon		
cg12822242		1	37	8	1.28E+08				chr8:1275€	S_Shelf
cg12877889		1	37	3	21446874	VENTXP7	NR_002311	TSS1500		
cg12934366		2	37	8	1960466				chr8:1956S	S_Shelf
cg13016524		1	37	11	16628944				chr11:1662	Island
cg13066016		1	37	1	63786083				chr1:63782	Island
cg13074470		1	37	3	1.27E+08				chr3:12685	Island
cg13112361	1		37	11	63258739	HRASLS5;HRASLS5;HRASLS5	NM_001146728;NM_001146729;NM_(	TSS200;TSS200;TSS200	chr11:6325	Island
cg13130934	1		37	15	25449922	SNORD115-21;SNORD115-20;SNORD115-15	NR_003313;NR_003312;NR_003307	TSS1500;TSS1500;TSS1500		
cg13193962		2	37	4	1.57E+08	CTSO	NM_001334	TSS200	chr4:15687	S_Shore
cg13209388		1	37	1	6355060	ACOT7;ACOT7;ACOT7;ACOT7	NM_181864;NM_181865;NM_007274;	Body;Body;Body;Body		
cg13229233	1		37	17	67326602				chr17:6732	S_Shelf
cg13229345	1		37	4	1.88E+08				chr4:1879E	Island
cg13230424	2		37	17	45930033	SP6	NM_199262	5'UTR	chr17:4592	S_Shore
cg13230424		3	37	17	45930033	SP6	NM_199262	5'UTR	chr17:4592	S_Shore
cg13279956		1	37	11	55653268	SPRYD5	NM_032681	Body		
cg13286318		1	37	8	57906058	IMPAD1	NM_017813	1stExon	chr8:57905	Island
cg13306164	1		37	1	33352184	HPCA	NM_002143	5'UTR	chr1:33351	Island
cg13312403	1		37	8	71246470	NCOA2	NM_006540	5'UTR		
cg13401339	1		37	2	24272928	FKBP1B;FKBP1B	NM_054033;NM_004116	Body;Body	chr2:24272	Island
cg13411336		1	37	11	43968842				chr11:439€	S_Shelf
cg13424673		2	37	1	50489744	AGBL4	NM_032785	TSS200	chr1:5048S	Island
cg13426079		3	37	1	41284948	KCNQ4;KCNQ4	NM_004700;NM_172163	Body;Body	chr1:41284	Island

cg13462158	3		37	1	55522104	PCSK9	NM_174936	Body		
cg13473576		1	37	7	97601586	MGC72080	NR_002822	Body	chr7:97601	Island
cg13488605	2		37	19	38908709	RASGRP4;RASGRP4;RASGRP4;RASGRP4;RASGR	NM_001146205;NM_001146207;NM_(	Body;Body;Body;Body;	chr19:3890	Island
cg13523386	1		37	4	71457838	AMBN	NM_016519	TSS200		
cg13560904		1	37	1	1.5E+08	OTUD7B	NM_020205	TSS200	chr1:14998	Island
cg13565382	1		37	10	63628787					
cg13565382		4	37	10	63628787					
cg13568258		1	37	19	48823427	CCDC114	NM_144577	TSS200	chr19:4882	N_Shore
cg13605988	1		37	15	45421612	DUOX1;DUOX1;DUOXA1	NM_175940;NM_017434;NM_144565	TSS1500;TSS1500;5'UT	chr15:4542	Island
cg13612295		1	37	18	72124791	FAM69C	NM_001044369	TSS1500	chr18:7212	S_Shore
cg13614440	2		37	17	76471012	DNAH17	NM_173628	Body	chr17:7647	N_Shore
cg13648344	1		37	9	36328161					
cg13663218		1	37	12	72666976	LOC283392;TRHDE;LOC283392	NR_026837;NM_013381;NR_026836	Body;1stExon;Body	chr12:7266	Island
cg13667739	1		37	14	1.06E+08	CRIP2	NM_001312	Body	chr14:1059	Island
cg13667739		1	37	14	1.06E+08	CRIP2	NM_001312	Body	chr14:1059	Island
cg13684188		1	37	9	1.35E+08	TTF1	NM_007344	TSS200	chr9:13528	Island
cg13686622		1	37	13	41634589	WBP4	NM_007187	TSS1500	chr13:4163	Island
cg13857186		1	37	9	1.16E+08	C9orf43;POLE3;C9orf43;POLE3	NM_152786;NR_027261;NM_152786;N	5'UTR;TSS1500;1stExon	chr9:11617	S_Shore
cg13861904	1		37	9	1.39E+08	NOTCH1	NM_017617	Body	chr9:13942	N_Shore
cg13892902		1	37	15	43478040	CCNDBP1;CCNDBP1;CCNDBP1;CCNDBP1	NM_037370;NM_012142;NR_027513;N	TSS200;Body;Body;Bod	chr15:4347	S_Shore
cg13896879	1		37	1	1.51E+08	ARNT;ARNT;ARNT	NM_178427;NM_001668;NM_178426	Body;Body;Body		
cg13918350	1		37	7	1153507	C7orf50;C7orf50;C7orf50	NM_001134395;NM_032350;NM_0011	Body;Body;Body	chr7:11553	N_Shore
cg13918350		1	37	7	1153507	C7orf50;C7orf50;C7orf50	NM_001134395;NM_032350;NM_0011	Body;Body;Body	chr7:11553	N_Shore
cg13944505		1	37	1	1.87E+08					
cg13995006	1		37	8	1.19E+08	MED30	NM_080651	TSS200	chr8:11853	N_Shore
cg13999514		1	37	7	1.06E+08	FLJ36031	NM_175884	TSS1500	chr7:10630	S_Shore
cg14022105	2		37	11	78288793				chr11:7828	S_Shelf
cg14022105		3	37	11	78288793				chr11:7828	S_Shelf
cg14048880		1	37	9	1.39E+08				chr9:13906	Island
cg14056644		1	37	4	1.12E+08	PITX2;PITX2	NM_153427;NM_153426	TSS1500;TSS1500	chr4:11156	N_Shore
cg14097440		1	37	14	74251018	C14orf43	NM_001043318	5'UTR	chr14:7425	N_Shelf
cg14109799	2		37	14	31495427	AP4S1;STRN3;STRN3;STRN3;STRN3;AP4S1	NM_007077;NM_014574;NM_014574;	5'UTR;1stExon;5'UTR;1	chr14:3149	Island
cg14157578		1	37	9	1.11E+08					
cg14185808		1	37	2	1.06E+08	MRPS9	NM_182640	TSS1500	chr2:10565	N_Shore
cg14200609		1	37	12	94853950	CCDC41;CCDC41;LOC144486	NM_016122;NM_001042399;NR_0270	TSS200;TSS200;Body	chr12:9485	Island
cg14237674	1		37	17	43239170	HEXIM2	NM_144608	5'UTR	chr17:4323	Island
cg14249856		1	37	7	77167329	PTPN12;PTPN12;PTPN12	NM_001131009;NM_001131008;NM_(	TSS200;TSS200;Body	chr7:77166	Island
cg14279842	2		37	14	50159788	KLHDC1	NM_172193	TSS200	chr14:5015	Island
cg14280905		5	37	9	1.36E+08	ADAMTSL2;ADAMTSL2	NM_014694;NM_001145320	Body;Body	chr9:13642	N_Shore
cg14294250		3	37	17	1957154	HIC1	NM_006497	TSS1500	chr17:1952	Island
cg14314896	1		37	19	430690	SHC2	NM_012435	Body	chr19:4292	S_Shore
cg14330460	4		37	6	1.69E+08					
cg14330460		7	37	6	1.69E+08					
cg14351127	1		37	9	1.03E+08	INVS;INVS	NM_014425;NM_183245	Body;Body		
cg14361033		1	37	9	1.25E+08	LHX6;LHX6	NM_199160;NM_014368	Body;Body	chr9:12498	N_Shore
cg14397696		1	37	13	1.06E+08					
cg14414764	1		37	8	67741966	SGK3;SGK3;SGK3	NM_170709;NM_001033578;NM_0132	Body;Body;Body		
cg14447152	1		37	9	1.4E+08	COBRA1	NM_015456	Body	chr9:14014	S_Shelf
cg14447152		2	37	9	1.4E+08	COBRA1	NM_015456	Body	chr9:14014	S_Shelf
cg14455170	1		37	13	42002398					

cg14466759	1		37	7	1.02E+08	CUX1;CUX1;CUX1	NM_181500;NM_181552;NM_001913	Body;Body;Body		
cg14466759		1	37	7	1.02E+08	CUX1;CUX1;CUX1	NM_181500;NM_181552;NM_001913	Body;Body;Body		
cg14467066	1		37	17	79419739	BAHCC1	NM_001080519	Body	chr17:7942	N_Shore
cg14482093		1	37	8	22461488	KIAA1967;C8orf58;KIAA1967	NM_021174;NM_001013842;NM_1992	TSS1500;3'UTR;TSS150	chr8:22462	N_Shore
cg14540650	1		37	9	6683274				chr9:66808	S_Shore
cg14543412	1		37	6	26224100	HIST1H3E	NM_003532	TSS1500	chr6:26225	N_Shore
cg14555127		3	37	7	35841578	SEPT7;SEPT7	NM_001011553;NM_001788	Body;Body	chr7:35840	Island
cg14616423	7		37	9	16315271					
cg14616423		1	37	9	16315271					
cg14639163	1		37	2	2.2E+08	WNT6	NM_006522	Body	chr2:21973	Island
cg14657159		2	37	12	56211136	ORMDL2;SARNP;SARNP;SARNP	NM_014182;NR_026723;NR_026722;N	TSS1500;Body;Body;Bo	chr12:5621	N_Shore
cg14710040	4		37	19	3985464	EEF2	NM_001961	TSS200	chr19:3984	Island
cg14737877		1	37	8	664783	ERICH1	NM_207332	Body	chr8:66399	Island
cg14780600	1		37	2	2.4E+08	HDAC4	NM_006037	5'UTR		
cg14795305		2	37	7	1.51E+08	SMARCD3;SMARCD3	NM_001003802;NM_003078	TSS1500;TSS1500	chr7:15097	Island
cg14834675		1	37	12	70083105	BEST3;BEST3	NM_032735;NM_152439	Body;TSS200		
cg14857851	1		37	3	15115139	ZFYVE20	NM_022340	3'UTR	chr3:1511€	N_Shore
cg14858469		1	37	15	96874050	NR2F2;NR2F2	NM_021005;NM_001145155	TSS200;Body	chr15:9687	Island
cg14866032	1		37	15	98195808				chr15:9819	N_Shore
cg14870156		1	37	6	33048540	HLA-DPB1	NM_002121	Body	chr6:33048	Island
cg14871601	1		37	7	64030039				chr7:64029	Island
cg14877741		1	37	9	1.26E+08	RABGAP1	NM_012197	TSS200	chr9:1257C	Island
cg14938587		1	37	7	1.56E+08					
cg14965968		1	37	3	44803235	KIF15;KIF15;KIAA1143	NM_020242;NM_020242;NM_020696	1stExon;5'UTR;TSS200	chr3:44802	Island
cg15002362	1		37	6	1.3E+08	LAMA2;LAMA2	NM_001079823;NM_000426	Body;Body		
cg15002362		1	37	6	1.3E+08	LAMA2;LAMA2	NM_001079823;NM_000426	Body;Body		
cg15002580	1		37	8	98636815				chr8:98636	Island
cg15025054		2	37	1	2.13E+08	TATDN3;NSL1;TATDN3;NSL1;TATDN3;TATDN3;	NM_001146171;NM_015471;NM_001C	TSS1500;1stExon;TSS15	chr1:21296	Island
cg15109571	2		37	10	1.04E+08	SUFU	NM_016169	1stExon	chr10:1042	Island
cg15119076		1	37	19	19976606	ZNF253	NM_021047	TSS200		
cg15140902		1	37	6	21667815	FLJ22536	NR_015410	Body	chr6:21665	S_Shore
cg15196058		1	37	4	1.31E+08					
cg15228928	2		37	13	1.13E+08				chr13:1127	Island
cg15330255		1	37	10	1.03E+08	FBXW4	NM_022039	TSS1500	chr10:1034	S_Shore
cg15333277	2		37	8	41733931	ANK1	NM_001142446	Body	chr8:41733	S_Shore
cg15347484	2		37	2	79515194					
cg15490897		1	37	1	55038020	ACOT11;ACOT11	NM_015547;NM_147161	Body;Body		
cg15518950		1	37	5	72415770	TMEM171;TMEM171	NM_001161342;NM_173490	TSS1500;TSS1500	chr5:72415	Island
cg15554438	1		37	11	92931280	SLC36A4	NM_152313	TSS200	chr11:9293	Island
cg15590056		1	37	7	1.5E+08					
cg15595333	1		37	11	1.18E+08					
cg15602420	1		37	1	20205059				chr1:20208	N_Shelf
cg15651394		1	37	2	36584981	CRIM1	NM_016441	Body	chr2:36584	Island
cg15681626	1		37	11	64739253				chr11:6473	Island
cg15685834		1	37	1	18970180	PAX7;PAX7;PAX7	NM_013945;NM_002584;NM_0011352	Body;Body;Body	chr1:18969	Island
cg15705470	1		37	5	87972265	LOC645323;LOC645323	NR_024383;NR_015436	Body;Body	chr5:87973	N_Shore
cg15709065		1	37	3	1.34E+08	AMOTL2	NM_016201	5'UTR	chr3:13409	N_Shore
cg15714041	1		37	9	32384344	ACO1	NM_002197	TSS1500	chr9:32384	N_Shore
cg15745910		1	37	13	1.13E+08					
cg15750986	1		37	7	98249103	NPTX2	NM_002523	Body	chr7:98245	S_Shore

cg15760840	1		37	7	27225222	HOXA11AS;HOXA11		NR_002795;NM_005523	Body;TSS1500	chr7:27225	Island
cg15810744		1	37	11	67211300	CORO1B;CORO1B		NM_001018070;NM_020441	TSS200;TSS1500		
cg15812873		1	37	16	28635015	SULT1A1		NM_177536	TSS200	chr16:2863	Island
cg15840462		1	37	1	969744	AGRN		NM_198576	Body	chr1:96796	Island
cg15846886	1		37	17	79790116	FAM195B;FAM195B		NM_207368;NM_001093767	5'UTR;5'UTR	chr17:7979	N_Shore
cg15877233	1		37	7	40611688	C7orf10		NM_024728	Body		
cg15892650		1	37	5	1.51E+08	ANXA6;ANXA6		NM_001155;NM_004033	Body;Body		
cg15913680		3	37	11	72353343	PDE2A;PDE2A;PDE2A;PDE2A		NM_001143839;NR_026572;NM_0011	1stExon;Body;Body;Bo	chr11:7235	Island
cg15917344	1		37	7	89975873	GTPBP10;GTPBP10		NM_001042717;NM_033107	TSS200;TSS200	chr7:89975	Island
cg15959921	1		37	4	8702279						
cg15974085	1		37	18	71815641	C18orf55;FBXO15;FBXO15		NM_014177;NM_152676;NM_001142	TSS200;TSS1500;TSS15	chr18:7181	Island
cg15974085		15	37	18	71815641	C18orf55;FBXO15;FBXO15		NM_014177;NM_152676;NM_001142	TSS200;TSS1500;TSS15	chr18:7181	Island
cg15994467	1		37	12	52401523	GRASP		NM_181711	Body	chr12:5240	Island
cg16000637		1	37	7	1274142	UNCX		NM_001080461	Body	chr7:12691	Island
cg16001460	3		37	6	57181802	PRIM2		NM_000947	TSS1500		
cg16001460		8	37	6	57181802	PRIM2		NM_000947	TSS1500		
cg16009381	2		37	5	1.73E+08	BOD1;BOD1;BOD1;BOD1		NM_138369;NM_138369;NM_001159	5'UTR;1stExon;5'UTR;1	chr5:17304	Island
cg16009381		1	37	5	1.73E+08	BOD1;BOD1;BOD1;BOD1		NM_138369;NM_138369;NM_001159	5'UTR;1stExon;5'UTR;1	chr5:17304	Island
cg16013630		2	37	1	1406606	ATAD3B		NM_031921	TSS1500	chr1:14068	N_Shore
cg16026088	1		37	9	1.33E+08	TOR1B		NM_014506	TSS200	chr9:13256	N_Shore
cg16060163		3	37	17	80454814					chr17:8045	Island
cg16084014		1	37	7	1.15E+08	MDFIC;MDFIC		NM_199072;NM_001166345	Body;Body		
cg16091269	1		37	3	1.97E+08					chr3:19718	N_Shore
cg16091269		1	37	3	1.97E+08					chr3:19718	N_Shore
cg16101346		1	37	1	1.87E+08	PTGS2		NM_000963	TSS1500	chr1:18664	S_Shore
cg16101636	1		37	6	29711438	LOC285830;LOC285830		NR_026972;NR_026973	Body;Body		
cg16101636		1	37	6	29711438	LOC285830;LOC285830		NR_026972;NR_026973	Body;Body		
cg16121685		5	37	1	1.62E+08	UHMK1		NM_175866	TSS200	chr1:16246	Island
cg16143319		1	37	2	32230813	MEMO1;MEMO1		NM_015955;NM_001137602	Body;Body	chr2:32234	N_Shelf
cg16208269		1	37	3	45665937	LIMD1		NM_014240	Body		
cg16226310	1		37	5	843633	ZDHHC11		NM_024786	Body	chr5:84345	Island
cg16226310		1	37	5	843633	ZDHHC11		NM_024786	Body	chr5:84345	Island
cg16233210	1		37	3	1.72E+08	FNDC3B;FNDC3B		NM_022763;NM_001135095	5'UTR;5'UTR		
cg16234298		1	37	22	17091297	psiTPTE22		NR_001591	Body	chr22:1709	S_Shore
cg16271221	1		37	3	1.89E+08						
cg16315155		1	37	19	55249794	KIR2DL3;KIR2DL3		NM_015868;NM_014511	TSS200;TSS200		
cg16348316	1		37	2	1.28E+08	PROC		NM_000312	Body	chr2:12818	Island
cg16349612	1		37	7	50849723	GRB10		NM_001001555	5'UTR	chr7:50849	N_Shore
cg16355305		1	37	12	65220152					chr12:6521	S_Shore
cg16404784	1		37	14	38087605					chr14:3809	N_Shelf
cg16410115	2		37	1	24240000	CNR2		NM_001841	TSS200		
cg16410115		1	37	1	24240000	CNR2		NM_001841	TSS200		
cg16459519	1		37	10	46970584	SYT15;SYT15;SYT15;SYT15		NM_031912;NM_181519;NM_031912;	1stExon;5'UTR;5'UTR;1	chr10:4696	Island
cg16481280	1		37	6	32120955	PPT2;PRRT1;PPT2		NM_005155;NM_030651;NM_138717	TSS1500;TSS1500;TSS1	chr6:32121	N_Shore
cg16506566	1		37	5	10420680		43530	NM_005885	Body		
cg16517838		1	37	14	1.06E+08					chr14:1061	S_Shore
cg16542426		1	37	15	23205622	WHAMML1		NR_003521	Body	chr15:2320	N_Shelf
cg16573957		1	37	8	21970273					chr8:21966	S_Shelf
cg16619071	1		37	19	52408324	ZNF649		NM_023074	TSS200		
cg16626809	1		37	21	46825856	COL18A1		NM_130445	Body	chr21:4682	Island

cg16668042	1		37	5	1.32E+08	HSPA4	NM_002154	Body	chr5:13238	Island
cg16668042		1	37	5	1.32E+08	HSPA4	NM_002154	Body	chr5:13238	Island
cg16681830		1	37	20	62586920	UCKL1AS;UCKL1	NR_027287;NM_017859	Body;Body	chr20:6258	Island
cg16707641		1	37	2	1.75E+08	WIPF1;WIPF1	NM_003387;NM_001077269	Body;Body		
cg16730825	1		37	11	928439	AP2A2	NM_012305	Body	chr11:9251	S_Shelf
cg16788797		1	37	8	75132833					
cg16790849	5		37	6	31829511	NEU1	NM_000434	Body	chr6:3183C	N_Shore
cg16790849		3	37	6	31829511	NEU1	NM_000434	Body	chr6:3183C	N_Shore
cg16837441	1		37	20	43935222	MATN4;MATN4;MATN4;RBPJL	NM_003833;NM_030592;NM_030590;	5'UTR;5'UTR;5'UTR;TSS	chr20:4393	Island
cg16872520		1	37	11	72929124	P2RY2;P2RY2;P2RY2	NM_176071;NM_176072;NM_002564	TSS1500;TSS1500;TSS1	chr11:7292	Island
cg17001464	1		37	7	1.31E+08	FLJ43663;FLJ43663	NR_015431;NR_024153	Body;Body		
cg17010112		1	37	4	77227123	STBD1	NM_003943	TSS1500	chr4:77227	N_Shore
cg17038667	1		37	11	69632883	FGF3	NM_005247	Body	chr11:6963	Island
cg17049418		1	37	6	3722453					
cg17278466		2	37	17	76798203	USP36	NM_025090	Body	chr17:7679	Island
cg17310773	4		37	22	41215357	SLC25A17;SLC25A17	NM_006358;NM_006358	1stExon;5'UTR	chr22:4121	Island
cg17310773		1	37	22	41215357	SLC25A17;SLC25A17	NM_006358;NM_006358	1stExon;5'UTR	chr22:4121	Island
cg17319849		1	37	1	1.55E+08	GBA;GBA;GBA;GBA	NM_001005749;NM_001005750;NM_(	TSS200;TSS200;TSS200;TSS200		
cg17330305	1		37	17	56834361	PPM1E	NM_014906	Body	chr17:5683	S_Shore
cg17330305		2	37	17	56834361	PPM1E	NM_014906	Body	chr17:5683	S_Shore
cg17351882	1		37	17	4692162	GLTPD2	NM_001014985	TSS200	chr17:4692	N_Shore
cg17392018	1		37	7	1.35E+08	CALD1;CALD1;CALD1;CALD1;CALD1	NM_033139;NM_033157;NM_033140;	Body;Body;Body;Body;Body		
cg17393572		1	37	5	1.76E+08	HK3	NM_002115	TSS200		
cg17419597		1	37	1	1.56E+08	BGLAP	NM_199173	TSS1500	chr1:15621	N_Shelf
cg17514665	1		37	17	1657533	SERPINF2;SERPINF2;SERPINF2	NM_000934;NM_001165921;NM_0011	Body;Body;Body	chr17:1657	Island
cg17599471	1		37	1	25174745				chr1:25174	Island
cg17656474		1	37	12	176424	IQSEC3	NM_001170738	1stExon	chr12:1756	S_Shore
cg17690515		2	37	19	1360278	MUM1;MUM1	NR_024247;NM_032853	Body;Body	chr19:136C	Island
cg17745251	11		37	10	21806139	C10orf140	NM_207371	Body	chr10:218C	Island
cg17745251		7	37	10	21806139	C10orf140	NM_207371	Body	chr10:218C	Island
cg17747332	1		37	19	11071239	SMARCA4;SMARCA4;SMARCA4	NM_003072;NM_001128849;NM_0011	TSS1500;TSS1500;TSS1	chr19:1107	Island
cg17749356		1	37	2	1.19E+08	CCDC93	NM_019044	Body		
cg17775382	1		37	5	1584233	SDHAP3	NR_003263	Body		
cg17782025	1		37	3	23958698	NKIRAS1;RPL15	NM_020345;NM_002948	TSS200;5'UTR	chr3:23957	Island
cg17794241	1		37	15	74658653	CYP11A1;CYP11A1	NM_000781;NM_001099773	Body;TSS200	chr15:7465	S_Shore
cg17815035		1	37	18	54790380				chr18:5478	S_Shore
cg17877237	2		37	13	32497491	EEF1DP3	NR_027062	Body		
cg17892069	2		37	7	70061616	AUTS2;AUTS2	NM_015570;NM_001127231	Body;Body	chr7:7006C	S_Shore
cg17892069		3	37	7	70061616	AUTS2;AUTS2	NM_015570;NM_001127231	Body;Body	chr7:7006C	S_Shore
cg17897445		1	37	8	7537473				chr8:75376	N_Shore
cg17918201	1		37	10	27150106	ABI1;ABI1;ABI1;ABI1	NM_005470;NM_001012752;NM_001(	TSS200;TSS200;TSS200	chr10:2714	Island
cg17956079	10		37	11	73358572	PLEKHB1;PLEKHB1;PLEKHB1;PLEKHB1;PLEKHB1	NM_001130035;NM_001130036;NM_(	TSS200;TSS1500;TSS200;Body;Body		
cg17956079		4	37	11	73358572	PLEKHB1;PLEKHB1;PLEKHB1;PLEKHB1;PLEKHB1	NM_001130035;NM_001130036;NM_(	TSS200;TSS1500;TSS200;Body;Body		
cg17987505		1	37	5	1.79E+08	SQSTM1;SQSTM1	NM_001142298;NM_001142299	5'UTR;5'UTR	chr5:17924	N_Shore
cg18007959	1		37	5	1.54E+08				chr5:15386	S_Shore
cg18056133	1		37	11	60609408	CCDC86	NM_024098	TSS200	chr11:606C	Island
cg18099523		1	37	6	1.46E+08	SHPRH;SHPRH	NM_173082;NM_001042683	TSS1500;TSS1500	chr6:14628	Island
cg18111500	3		37	14	61655727					
cg18111500		10	37	14	61655727					
cg18193219		1	37	3	53164697	RFT1	NM_052859	TSS1500	chr3:53164	Island

cg18207011	1		37	14	68861095	RAD51L1;RAD51L1;RAD51L1	NM_133509;NM_002877;NM_133510	Body;Body;Body		
cg18217175	2		37	1	18967020	PAX7;PAX7;PAX7	NM_013945;NM_002584;NM_0011352	Body;Body;Body	chr1:18967	N_Shore
cg18217175		1	37	1	18967020	PAX7;PAX7;PAX7	NM_013945;NM_002584;NM_0011352	Body;Body;Body	chr1:18967	N_Shore
cg18220799	1		37	18	44099340	LOXHD1;LOXHD1;LOXHD1	NM_001145473;NM_144612;NM_0011	TSS1500;Body;Body		
cg18239858		1	37	7	1.02E+08	CUX1;CUX1;CUX1	NM_181500;NM_181552;NM_001913	Body;Body;Body		
cg18325160	1		37	3	1.93E+08	OPA1;OPA1;OPA1;OPA1;OPA1;OPA1;OPA1;OP.	NM_130836;NM_130831;NM_130837;	5'UTR;5'UTR;5'UTR;1st	chr3:19331	Island
cg18337963	1		37	11	46383209	DGKZ;DGKZ;DGKZ;DGKZ;DGKZ	NM_001105540;NM_201533;NM_0011	1stExon;Body;5'UTR;Body;Body		
cg18391209		1	37	1	2.24E+08	CAPN8	NM_001143962	Body	chr1:22374	S_Shelf
cg18415822		1	37	7	1.56E+08				chr7:15585	S_Shore
cg18452149	1		37	3	1.08E+08	KIAA1524;DZIP3;KIAA1524	NM_020890;NM_014648;NM_020890	5'UTR;TSS200;1stExon	chr3:1083C	Island
cg18460938		1	37	15	86842630	AGBL1	NM_152336	Body		
cg18496140	1		37	11	44286375	ALX4	NM_021926	3'UTR		
cg18501555		1	37	4	1.12E+08	PITX2;PITX2	NM_153427;NM_153426	5'UTR;5'UTR	chr4:11155	Island
cg18561676	2		37	1	1.49E+08				chr1:14885	Island
cg18561676		4	37	1	1.49E+08				chr1:14885	Island
cg18580296	1		37	7	15726411	MEOX2	NM_005924	TSS200		
cg18580296		2	37	7	15726411	MEOX2	NM_005924	TSS200		
cg18633379		1	37	18	59855274	PIGN;PIGN;KIAA1468	NM_012327;NM_176787;NM_020854	TSS1500;TSS1500;Body	chr18:5985	S_Shore
cg18633561		1	37	7	1.22E+08	FEZF1;FEZF1	NM_001160264;NM_001024613	Body;Body	chr7:12194	N_Shore
cg18676488	1		37	5	70848105	BDP1	NM_018429	Body		
cg18676488		2	37	5	70848105	BDP1	NM_018429	Body		
cg18686165		1	37	3	57200492	IL17RD	NM_017563	TSS1500	chr3:57198	S_Shore
cg18700813		3	37	14	1.03E+08	RAGE	NM_014226	Body		
cg18710412	1		37	5	90654191					
cg18724069		1	37	5	39721760				chr5:39721	Island
cg18762036		1	37	2	1.08E+08					
cg18779296	1		37	4	41878119				chr4:4188C	N_Shelf
cg18855674		1	37	8	72469553				chr8:72468	Island
cg18862888	4		37	10	31346162					
cg18902057		1	37	16	88636444	ZC3H18	NM_144604	TSS1500	chr16:8863	Island
cg18927185	1		37	6	33091634	HLA-DPB2	NR_001435	Body		
cg18954388		1	37	11	20621495	SLC6A5	NM_004211	Body	chr11:2062	N_Shore
cg19037304		2	37	7	19812760	TMEM196	NM_152774	TSS1500	chr7:19812	Island
cg19132477	1		37	3	8484313					
cg19132477		1	37	3	8484313					
cg19154754	1		37	11	48977527					
cg19157696	1		37	11	27384984	CCDC34;CCDC34	NM_080654;NM_030771	TSS200;TSS200	chr11:2738	S_Shore
cg19190016	1		37	17	81025684				chr17:8102	S_Shore
cg19207486	1		37	19	22323107				chr19:2232	S_Shelf
cg19227053	1		37	20	60116795	CDH4	NM_001794	Body	chr20:6011	N_Shelf
cg19265948	2		37	12	23229286					
cg19265948		1	37	12	23229286					
cg19327615	1		37	20	19955436	RIN2	NM_018993	Body	chr20:1995	N_Shore
cg19327615		1	37	20	19955436	RIN2	NM_018993	Body	chr20:1995	N_Shore
cg19380675	1		37	22	32808489	C22orf28	NM_014306	TSS1500	chr22:3280	S_Shore
cg19409687		1	37	11	65682960				chr11:6568	N_Shelf
cg19441646		1	37	14	1.03E+08	RAGE	NM_014226	3'UTR	chr14:1026	Island
cg19448318		2	37	6	30698784	FLOT1	NM_005803	Body		
cg19469087		1	37	7	98556941	TRRAP	NM_003496	Body	chr7:98552	S_Shelf
cg19480274		1	37	20	37098327				chr20:371C	N_Shelf

cg19484381		1	37	6	28890673	TRIM27	NM_006510	Body	chr6:2889(	N_Shore
cg19488906		1	37	6	1.62E+08	AGPAT4	NM_020133	5'UTR		
cg19513744		2	37	3	56836209	ARHGEF3;ARHGEF3	NM_001128615;NM_019555	Body;TSS1500	chr3:56835	Island
cg19514542		1	37	10	1.26E+08	CPXM2	NM_198148	Body	chr10:1256	N_Shelf
cg19559392	7		37	2	39103372	MORN2;DHX57	NM_001145450;NM_198963	5'UTR;TSS1500	chr2:39102	S_Shore
cg19562312	1		37	10	44069847	ZNF239;ZNF239;ZNF239	NM_001099284;NM_001099282;NM_(	5'UTR;5'UTR;5'UTR	chr10:4406	Island
cg19625088	1		37	5	87976156	LOC645323	NR_015436	Body	chr5:87976	Island
cg19627869		1	37	12	56391069	SUOX;SUOX;SUOX;SUOX;SUOX;SUOX	NM_001032386;NM_001032387;NM_(	5'UTR;1stExon;1stExon;5'UTR;5'UTR;1stExon		
cg19693446	1		37	14	1.02E+08					
cg19749898	1		37	11	1712765	HCCA2	NM_053005	Body	chr11:1715	N_Shelf
cg19777001	1		37	14	1.03E+08	RCOR1	NM_015156	Body		
cg19837938	1		37	5	23507458	PRDM9	NM_020227	TSS1500		
cg19837938		2	37	5	23507458	PRDM9	NM_020227	TSS1500		
cg19852211	1		37	5	1.4E+08	PCDHA2;PCDHA1;PCDHA1;PCDHA4;PCDHA3;P(	NM_018905;NM_031411;NM_018900;	Body;Body;Body;1stEx(	chr5:14018	Island
cg19852211		1	37	5	1.4E+08	PCDHA2;PCDHA1;PCDHA1;PCDHA4;PCDHA3;P(	NM_018905;NM_031411;NM_018900;	Body;Body;Body;1stEx(	chr5:14018	Island
cg19855573	2		37	1	1.52E+08	FLG	NM_002016	TSS200		
cg19865375		1	37	7	86629801	KIAA1324L;KIAA1324L	NM_001142749;NR_030672	Body;Body		
cg19875976	1		37	9	1.14E+08	KIAA0368	NM_001080398	Body	chr9:11424	Island
cg19875976		1	37	9	1.14E+08	KIAA0368	NM_001080398	Body	chr9:11424	Island
cg19991948	1		37	10	1.21E+08	TIAL1;TIAL1	NM_001033925;NM_003252	3'UTR;3'UTR		
cg19998654		1	37	2	37458983	CEBPZ;C2orf56;C2orf56	NM_005760;NM_144736;NM_001083(	TSS1500;Body;Body	chr2:37458	Island
cg20059151		1	37	1	95286311	SLC44A3;SLC44A3	NM_152369;NM_001114106	5'UTR;Body	chr1:95285	Island
cg20069765	1		37	6	33036464	HLA-DPA1	NM_033554	Body		
cg20081364	1		37	20	13202225	ISM1	NM_080826	TSS200	chr20:132(	Island
cg20155875	1		37	17	66452567	WIPI1	NM_017983	Body	chr17:6645	N_Shore
cg20162076	3		37	6	6588089	LY86;LOC285780	NM_004271;NR_026970	TSS1500;Body		
cg20162076		5	37	6	6588089	LY86;LOC285780	NM_004271;NR_026970	TSS1500;Body		
cg20172795		1	37	6	1.27E+08	RSPO3	NM_032784	Body	chr6:12744	N_Shore
cg20183094		4	37	7	1.22E+08				chr7:12195	Island
cg20200361		6	37	2	43775888	THADA;THADA	NM_022065;NM_001083953	Body;Body		
cg20268522		2	37	11	1.13E+08	NCAM1;NCAM1;NCAM1;NCAM1;NCAM1;NCAI	NM_001076682;NM_001076682;NM_(	1stExon;5'UTR;5'UTR;1	chr11:1128	N_Shore
cg20274430		1	37	22	41075992	MCHR1	NM_005297	Body		
cg20294319	1		37	13	47253842	LRCH1;LRCH1;LRCH1	NM_015116;NM_001164213;NM_0011	Body;Body;Body		
cg20319405		1	37	11	10830230	EIF4G2;EIF4G2	NM_001418;NM_001042559	5'UTR;5'UTR	chr11:1082	Island
cg20327784	1		37	17	73703307	SAP30BP	NM_013260	3'UTR		
cg20327784		1	37	17	73703307	SAP30BP	NM_013260	3'UTR		
cg20458353		2	37	7	21260293					
cg20500836		1	37	17	78818645	RPTOR;RPTOR	NM_001163034;NM_020761	Body;Body	chr17:7881	Island
cg20532937	1		37	10	13748985	FRMD4A	NM_018027	Body		
cg20555922	1		37	20	33880266	FAM83C	NM_178468	TSS200	chr20:3387	S_Shore
cg20587808	1		37	3	1.84E+08				chr3:18432	Island
cg20593868	1		37	20	62588672	UCKL1;ZNF512B	NM_017859;NM_020713	TSS1500;3'UTR	chr20:6258	S_Shore
cg20593868		1	37	20	62588672	UCKL1;ZNF512B	NM_017859;NM_020713	TSS1500;3'UTR	chr20:6258	S_Shore
cg20684251	3		37	19	51840554	VSIG10L	NM_001163922	Body	chr19:5184	N_Shore
cg20684251		1	37	19	51840554	VSIG10L	NM_001163922	Body	chr19:5184	N_Shore
cg20707630	1		37	15	25018449				chr15:2501	Island
cg20743744	1		37	4	1243849	C4orf42;CTBP1;CTBP1	NM_052861;NM_001328;NM_001012(	TSS1500;TSS1500;TSS1	chr4:12414	Island
cg20888142	1		37	3	12045662	SYN2;SYN2	NM_003178;NM_133625	TSS200;TSS200	chr3:12045	Island
cg20914725	1		37	2	74776831	LOXL3	NM_032603	Body	chr2:74776	Island
cg21000072	1		37	1	3567408	WDR8	NM_017818	TSS1500	chr1:35664	Island

cg21007262		1	37	20	18122863	PET117;CSRP2BP	NM_001164811;NM_020536	Body;TSS200	chr20:1811	S_Shelf
cg21102950		4	37	3	1.56E+08	C3orf33	NM_173657	TSS200	chr3:15552	Island
cg21117978		1	37	2	64957132					
cg21200923		1	37	18	9481257	RALBP1	NM_006788	5'UTR	chr18:9478	S_Shelf
cg21211367		1	37	2	1.62E+08				chr2:16209	N_Shore
cg21231400		2	37	5	1.55E+08				chr5:15510	Island
cg21234471	2		37	9	92023797	SEMA4D;SEMA4D	NM_001142287;NM_006378	5'UTR;5'UTR		
cg21242508		1	37	22	28199621				chr22:2819	S_Shore
cg21251791		1	37	2	3129858					
cg21263471	1		37	17	56399584	BZRAP1;BZRAP1	NM_004758;NM_024418	Body;Body	chr17:5640	N_Shelf
cg21314304	1		37	17	41135517	RUNDC1	NM_173079	Body	chr17:4113	S_Shelf
cg21434114		1	37	18	3450282	TGIF1;TGIF1;TGIF1;TGIF1;TGIF1;TGIF1;TGIF1;T(	NM_174886;NM_173208;NM_003244;	5'UTR;5'UTR;5'UTR;5'U	chr18:3448	Island
cg21441716	1		37	1	1.76E+08					
cg21450738		1	37	8	1.01E+08				chr8:10090	S_Shelf
cg21481322	1		37	8	40984072					
cg21503582	1		37	7	1.51E+08				chr7:15100	Island
cg21503582		1	37	7	1.51E+08				chr7:15100	Island
cg21549195	1		37	19	52452368				chr19:5245	Island
cg21552319	1		37	6	24356996	DCDC2;KAAG1	NM_016356;NM_181337	Body;TSS200	chr6:24357	N_Shore
cg21552319		2	37	6	24356996	DCDC2;KAAG1	NM_016356;NM_181337	Body;TSS200	chr6:24357	N_Shore
cg21584710		1	37	17	72306141	DNAI2	NM_023036	Body		
cg21584983	1		37	19	11640070	ECSIT;ECSIT;ECSIT;ECSIT	NM_001142464;NR_024551;NM_0011	TSS200;TSS1500;TSS20	chr19:1163	S_Shore
cg21584983		1	37	19	11640070	ECSIT;ECSIT;ECSIT;ECSIT	NM_001142464;NR_024551;NM_0011	TSS200;TSS1500;TSS20	chr19:1163	S_Shore
cg21590497	1		37	19	23299649				chr19:2329	N_Shore
cg21593588	1		37	4	1.1E+08	OSTC	NM_021227	Body	chr4:10957	S_Shelf
cg21643086	15		37	6	27243037					
cg21643086		12	37	6	27243037					
cg21686797	1		37	6	30303645	TRIM39;TRIM39	NM_172016;NM_021253	Body;Body		
cg21715599	1		37	15	92463554	SLCO3A1;SLCO3A1	NM_001145044;NM_013272	Body;Body	chr15:9245	S_Shelf
cg21729798		1	37	6	24357078	DCDC2;KAAG1	NM_016356;NM_181337	Body;TSS200	chr6:24357	N_Shore
cg21737039	1		37	4	8271391	HTRA3	NM_053044	TSS200	chr4:82712	Island
cg21737039		2	37	4	8271391	HTRA3	NM_053044	TSS200	chr4:82712	Island
cg21857885	3		37	14	60337864	RTN1	NM_021136	TSS1500	chr14:6033	S_Shore
cg21857885		2	37	14	60337864	RTN1	NM_021136	TSS1500	chr14:6033	S_Shore
cg21947488	2		37	16	2185289	PKD1;PKD1	NM_001009944;NM_000296	Body;Body	chr16:2185	Island
cg21947488		2	37	16	2185289	PKD1;PKD1	NM_001009944;NM_000296	Body;Body	chr16:2185	Island
cg22028161		1	37	2	95658954					
cg22078976	1		37	20	58515561	C20orf177;C20orf177;PPP1R3D	NM_022106;NM_022106;NM_006242	1stExon;5'UTR;TSS150(	chr20:5851	Island
cg22079902		1	37	5	23507644	PRDM9	NM_020227	TSS200		
cg22169384		2	37	12	1.29E+08	GLT1D1	NM_144669	Body		
cg22199410	1		37	12	51985016	SCN8A	NM_014191	TSS200	chr12:5198	Island
cg22205573	4		37	13	1.14E+08	LAMP1	NM_005561	Body		
cg22205573		1	37	13	1.14E+08	LAMP1	NM_005561	Body		
cg22240520	1		37	7	1.59E+08	VIPR2	NM_003382	Body	chr7:15885	N_Shore
cg22380533		1	37	19	55690741	SYT5	NM_003180	5'UTR	chr19:5569	Island
cg22384904		1	37	1	2.28E+08				chr1:22797	N_Shore
cg22389137	1		37	4	1.13E+08				chr4:11343	Island
cg22491379	2		37	2	1.21E+08	PTPN4	NM_002830	5'UTR		
cg22491379		1	37	2	1.21E+08	PTPN4	NM_002830	5'UTR		
cg22499893	1		37	1	24307535	SFRS13A;SFRS13A	NM_054016;NM_006625	TSS1500;TSS1500	chr1:24306	S_Shore

cg22510362	1		37	16	87251839				chr16:8725	Island
cg22630628	1		37	10	73337125	CDH23;CDH23	NM_052836;NM_022124	Body;Body		
cg22657457		1	37	5	75469969	SV2C	NM_014979	Body	chr5:75469	Island
cg22730967	1		37	1	2.03E+08	ADORA1;ADORA1	NM_001048230;NM_000674	5'UTR;5'UTR	chr1:20309	Island
cg22730967		1	37	1	2.03E+08	ADORA1;ADORA1	NM_001048230;NM_000674	5'UTR;5'UTR	chr1:20309	Island
cg22791936		1	37	6	1.37E+08	SLC35D3;SLC35D3	NM_001008783;NM_001008783	1stExon;5'UTR	chr6:13724	Island
cg22819135	1		37	5	41920822	C5orf51	NM_175921	3'UTR		
cg22821289	1		37	11	1.16E+08					
cg22827060	1		37	13	30424359	UBL3;UBL3	NM_007106;NM_007106	1stExon;5'UTR	chr13:3042	Island
cg22836434		1	37	11	86014570	C11orf73;C11orf73;C11orf73;C11orf73	NM_016401;NR_024597;NR_024596;N	Body;Body;Body;Body		
cg22856949		1	37	10	1.27E+08	C10orf137	NM_015608	TSS200	chr10:1274	Island
cg22877380		1	37	2	1.32E+08	ARHGEF4;ARHGEF4	NM_032995;NM_015320	3'UTR;3'UTR		
cg22963777	1		37	20	34115350	C20orf173;C20orf173	NM_001145350;NR_026933	3'UTR;Body		
cg23013894	1		37	5	5025912					
cg23067355		1	37	6	1.25E+08				chr6:12542	S_Shelf
cg23078194	1		37	6	27661566					
cg23146197		1	37	12	66271002	HMGA2;HMGA2	NM_003484;NM_003483	Body;Body		
cg23191118		1	37	8	1.46E+08	SCRT1	NM_031309	TSS1500	chr8:14555	Island
cg23210118		1	37	10	95653378	TMEM20;TMEM20	NM_153226;NM_001134658	TSS1500;TSS1500	chr10:9565	N_Shore
cg23226134		5	37	1	11866389	CLCN6;CLCN6;CLCN6;CLCN6;MTHFR	NM_001286;NM_021737;NM_021735;	1stExon;1stExon;1stExo	chr1:11865	Island
cg23232056	1		37	1	1.21E+08					
cg23239690	3		37	10	1.32E+08	MGMT	NM_002412	Body		
cg23239690		1	37	10	1.32E+08	MGMT	NM_002412	Body		
cg23275972	1		37	7	1.57E+08					
cg23283362		1	37	1	45263371				chr1:45265	N_Shelf
cg23285761	2		37	8	38089462	DDHD2;DDHD2;DDHD2	NM_015214;NM_001164234;NM_0011	5'UTR;5'UTR;TSS200	chr8:38088	Island
cg23350744	1		37	5	1.22E+08				chr5:12242	N_Shelf
cg23369529		1	37	1	68149989	GADD45A	NM_001924	TSS1500	chr1:68150	N_Shore
cg23442853		1	37	3	1.22E+08	PARP15;PARP15	NM_001113523;NM_001113523	5'UTR;1stExon	chr3:12229	N_Shore
cg23465289	1		37	5	54909384					
cg23509665		2	37	7	1.58E+08	PTPRN2;PTPRN2;PTPRN2	NM_002847;NM_130842;NM_130843	Body;Body;Body		
cg23517116		1	37	8	65499841				chr8:65499	Island
cg23574427		1	37	4	1.23E+08	BBS7;CCNA2	NM_176824;NM_001237	3'UTR;TSS1500	chr4:12274	S_Shore
cg23645639	1		37	19	21203403	ZNF430	NM_025189	TSS200		
cg23660155	1		37	1	22915626	EPHA8;EPHA8	NM_001006943;NM_020526	Body;Body		
cg23692620	1		37	19	19971862				chr19:1997	Island
cg23730260		1	37	11	8290409				chr11:8289	S_Shore
cg23777173		2	37	12	1.33E+08	GALNT9	NM_001122636	Body	chr12:1326	S_Shore
cg23802518		1	37	10	80827482	ZMIZ1;LOC283050;LOC283050;LOC283050	NM_020338;NR_024431;NR_024429;N	TSS1500;TSS1500;TSS1	chr10:8082	Island
cg23813156		1	37	20	47935243				chr20:4793	Island
cg23837438		1	37	10	1.35E+08				chr10:1351	Island
cg23951961	1		37	4	42154912	BEND4;BEND4	NM_207406;NM_001159547	TSS200;TSS200	chr4:42152	Island
cg23970785		1	37	16	11036526	DEXI	NM_014015	TSS1500	chr16:1103	Island
cg24030735	1		37	17	10533089	MYH3	NM_002470	Body		
cg24099067		1	37	6	26758750					
cg24177983		1	37	11	2398403	CD81	NM_004356	TSS200	chr11:2398	Island
cg24216966		1	37	6	31540121	LTA;LTA;LTA	NM_000595;NM_001159740;NM_0005	1stExon;5'UTR;5'UTR		
cg24243790		1	37	4	9760736					
cg24285811	1		37	9	1.39E+08				chr9:13914	Island
cg24300033		1	37	2	2.42E+08				chr2:24164	N_Shore

cg24330818		1	37	13	33002388	N4BP2L1;N4BP2L1	NM_052818;NM_001079691	TSS200;TSS200	chr13:3300	S_Shore
cg24364491	2		37	6	32806906	TAP2;TAP2	NM_018833;NM_000544	TSS1500;TSS1500	chr6:32806	S_Shore
cg24408706		2	37	11	363484					
cg24453123	1		37	15	35374148					
cg24453123		1	37	15	35374148					
cg24533904		1	37	1	1.84E+08	C1orf21	NM_030806	Body		
cg24541871		1	37	2	55845713	SMEK2;SMEK2	NM_001122964;NM_020463	TSS1500;TSS1500	chr2:55844	S_Shore
cg24547359		1	37	1	53975500	GLIS1	NM_147193	Body		
cg24550933	1		37	1	1.12E+08	LOC441897	NR_029429	TSS200		
cg24563317	1		37	8	48872822	MCM4;MCM4;PRKDC;PRKDC	NM_005914;NM_182746;NM_006904;	TSS1500;TSS1500;TSS2	chr8:48872	Island
cg24594224		1	37	6	33136015	COL11A2;COL11A2;COL11A2	NM_080679;NM_080681;NM_080680	Body;Body;Body		
cg24629356		1	37	1	35122551					
cg24706188		1	37	8	55088456					
cg24710435		1	37	10	14543847					
cg24734300	1		37	7	35677335	HERPUD2	NM_022373	Body		
cg24766104	1		37	19	16309072	AP1M1;AP1M1	NM_032493;NM_001130524	Body;Body	chr19:1630	Island
cg24766104		2	37	19	16309072	AP1M1;AP1M1	NM_032493;NM_001130524	Body;Body	chr19:1630	Island
cg24779831	1		37	2	2799370					
cg24794433	1		37	9	1.24E+08	DAB2IP	NM_032552	Body	chr9:12446	Island
cg24811352		1	37	5	1.41E+08	PCDHB6	NM_018939	TSS1500	chr5:14053	N_Shore
cg24839562	1		37	6	31673423	LY6G6F	NM_001003693	TSS1500	chr6:3167C	S_Shelf
cg24856050		1	37	19	3192433	NCLN	NM_020170	Body	chr19:3192	Island
cg24864663	1		37	15	43532243	TGM5;TGM5	NM_201631;NM_004245	Body;Body		
cg24891539	3		37	8	55370407	SOX17	NM_022454	TSS200	chr8:5537C	Island
cg24891539		1	37	8	55370407	SOX17	NM_022454	TSS200	chr8:5537C	Island
cg24917945	1		37	10	1.21E+08	C10orf46	NM_153810	TSS200	chr10:1205	Island
cg24968786		1	37	11	66360800	CCDC87;CCS	NM_018219;NM_005125	TSS1500;Body	chr11:6636	Island
cg25013910		1	37	2	1478219	TPO;TPO;TPO;TPO	NM_175719;NM_000547;NM_175721;	Body;Body;Body;Body	chr2:14807	N_Shelf
cg25025455	1		37	3	1.34E+08	CEP63;CEP63;ANAPC13;CEP63;CEP63;ANAPC13	NM_001042383;NM_001042400;NR_0	5'UTR;5'UTR;TSS200;5'	chr3:1342C	Island
cg25092989	1		37	8	10932494	XKR6	NM_173683	Body	chr8:10928	S_Shelf
cg25100962		2	37	12	31782808					
cg25124476	1		37	7	1.5E+08	TMEM176B;TMEM176B;TMEM176A;TMEM17(	NM_001101312;NM_001101314;NM_(	TSS200;TSS200;TSS200	chr7:15049	Island
cg25149069		2	37	1	2.33E+08	KIAA1804	NM_032435	1stExon	chr1:23346	Island
cg25154733		2	37	13	28965632	FLT1;FLT1;FLT1	NM_001159920;NM_001160030;NM_(	Body;Body;Body		
cg25161386		1	37	17	27621177	NUFIP2	NM_020772	TSS200	chr17:2762	Island
cg25181381	1		37	11	1.34E+08					
cg25193384	1		37	7	39837692				chr7:39833	S_Shelf
cg25194328		1	37	5	54472477				chr5:54468	S_Shelf
cg25206705		2	37	17	1082774	ABR;ABR	NM_021962;NM_001159746	Body;5'UTR	chr17:1082	Island
cg25236028		1	37	17	767334	NXN	NM_022463	Body		
cg25246158		1	37	19	1940184	CSNK1G2	NM_001319	TSS1500	chr19:194C	N_Shore
cg25259296		1	37	21	43235862	PRDM15;PRDM15	NM_022115;NM_001040424	Body;Body	chr21:4323	N_Shore
cg25328184	1		37	5	1.1E+08	TSLP	NM_033035	TSS1500	chr5:1104C	N_Shelf
cg25328184		1	37	5	1.1E+08	TSLP	NM_033035	TSS1500	chr5:1104C	N_Shelf
cg25334660		1	37	8	42270520				chr8:42268	S_Shore
cg25356214	2		37	12	51611871	POU6F1	NR_026893	TSS1500	chr12:5161	S_Shore
cg25367905		1	37	5	1.77E+08	RGS14	NM_006480	Body	chr5:17678	S_Shore
cg25370231		1	37	10	13391182	SEPHS1	NM_012247	TSS1500		
cg25402787		1	37	15	53084679				chr15:5308	N_Shore
cg25409448		2	37	19	49558849	CGB7;CGB7	NM_033142;NM_033142	5'UTR;1stExon	chr19:4955	N_Shore

cg25417551		1	37	17	35767582	TADA2A;TADA2A;ACACA;TADA2A;ACACA;TAD	NM_001488;NM_133439;NM_198834;	1stExon;1stExon;TSS15	chr17:3576	S_Shore
cg25427880		1	37	10	1.02E+08				chr10:1023	Island
cg25456593		1	37	11	70672858	SHANK2	NM_012309	Body	chr11:7067	Island
cg25502179		1	37	3	46875342	PRSS42	NM_182702	Body	chr3:46874	Island
cg25545088		1	37	6	43398446	ABCC10	NM_033450	TSS1500	chr6:43395	S_Shelf
cg25550629		1	37	12	53546895					
cg25714381		1	37	7	76751893	CCDC146	NM_020879	TSS200	chr7:7675C	S_Shore
cg25734864	2		37	13	1.14E+08	PROZ	NM_003891	1stExon		
cg25756867		1	37	3	1.8E+08					
cg25758828		1	37	2	1.14E+08	PAX8;PAX8;PAX8;PAX8;PAX8;LOC440839	NM_013953;NM_003466;NM_013952;	3'UTR;Body;3'UTR;Body;3'UTR;Body		
cg25778535		1	37	11	8190572	RIC3;RIC3;RIC3;RIC3	NM_001135109;NM_024557;NM_0011	1stExon;5'UTR;5'UTR;1	chr11:819C	Island
cg25803630	2		37	8	29885309				chr8:29884	Island
cg25820279	1		37	2	1.77E+08				chr2:1770C	S_Shore
cg25824218	1		37	12	25104798				chr12:251C	S_Shelf
cg25824218		1	37	12	25104798				chr12:251C	S_Shelf
cg25859012		1	37	2	2.2E+08	BCS1L;ZNF142;BCS1L	NM_004328;NM_001105537;NM_001C	TSS200;TSS200;TSS200	chr2:21952	Island
cg25884094		1	37	8	17785204	PCM1	NM_006197	5'UTR	chr8:1778C	S_Shelf
cg25894839		1	37	1	1.62E+08	FCRLB	NM_001002901	Body	chr1:1616S	Island
cg25943588		1	37	11	14993913	CALCA;CALCA;CALCA	NM_001033952;NM_001033953;NM_C	TSS200;TSS200;TSS200	chr11:1499	N_Shore
cg26003814		3	37	1	16444599					
cg26053864	1		37	15	35087635	ACTC1	NM_005159	5'UTR		
cg26074111	1		37	6	24776063	GMNN	NM_015895	5'UTR	chr6:24775	S_Shore
cg26084511		1	37	7	20818226				chr7:20817	Island
cg26091183	3		37	17	78387382	LOC100294362	NR_029376	Body	chr17:7838	N_Shore
cg26091183		2	37	17	78387382	LOC100294362	NR_029376	Body	chr17:7838	N_Shore
cg26105278		1	37	1	3624054	TP73;TP73;TP73;TP73	NM_001126240;NM_005427;NM_0011	Body;Body;Body;Body	chr1:36233	S_Shore
cg26114595	1		37	17	28395749	EFCAB5;EFCAB5;EFCAB5	NR_026738;NM_198529;NM_0011450	Body;Body;Body		
cg26129303		1	37	8	1.42E+08					
cg26148236	1		37	2	2.33E+08	EFHD1;EFHD1	NM_025202;NR_027663	TSS1500;Body	chr2:23349	Island
cg26149738		1	37	19	34287125	KCTD15;KCTD15;KCTD15	NM_001129994;NM_024076;NM_0011	TSS1500;TSS1500;TSS1	chr19:3428	Island
cg26163463	1		37	5	1.4E+08	EIF4EBP3;ANKHD1-EIF4EBP3	NM_003732;NM_020690	TSS1500;Body	chr5:13992	N_Shore
cg26203055		2	37	18	19284713	ABHD3;ABHD3	NM_138340;NM_138340	1stExon;5'UTR	chr18:1928	Island
cg26266950	2		37	7	19160621				chr7:19156	S_Shelf
cg26384229	9		37	12	38710491	ALG10B	NM_001013620	TSS200	chr12:3871	Island
cg26384229		14	37	12	38710491	ALG10B	NM_001013620	TSS200	chr12:3871	Island
cg26490274		1	37	16	4385435	GLIS2	NM_032575	Body		
cg26524638	1		37	11	2481449	KCNQ1;KCNQ1	NM_181798;NM_000218	TSS1500;Body		
cg26541533		1	37	6	1.64E+08				chr6:16425	Island
cg26573704		2	37	5	33892223	ADAMTS12	NM_030955	TSS200	chr5:33892	Island
cg26575535	1		37	16	65157839				chr16:6515	S_Shore
cg26586710	1		37	10	1.34E+08	PWWP2B;PWWP2B	NM_001098637;NM_138499	Body;Body	chr10:1342	N_Shore
cg26588943		2	37	4	1.73E+08	GALNTL6;GALNTL6	NM_001034845;NM_001034845	1stExon;5'UTR	chr4:17273	Island
cg26616283	1		37	7	11871535	THSD7A	NM_015204	1stExon		
cg26616283		1	37	7	11871535	THSD7A	NM_015204	1stExon		
cg26634911		2	37	4	47916863	NFXL1	NM_152995	TSS1500	chr4:47915	Island
cg26651514	1		37	13	33864734					
cg26654519		1	37	8	1.43E+08	FLJ43860	NM_207414	TSS200		
cg26712605		1	37	6	37745367					
cg26759552		2	37	7	1.4E+08	LOC100134229;JHDM1D	NR_024451;NM_030647	Body;TSS1500	chr7:13987	Island
cg26777456		1	37	12	54454250	FLJ12825	NR_026655	Body	chr12:5445	Island

cg26835440	2		37	8	55037773					
cg26919149	2		37	6	1.69E+08				chr6:16850	Island
cg26933136		1	37	1	28661842	MED18;MED18	NM_001127350;NM_017638	3'UTR;3'UTR		
cg26946235	2		37	10	1.35E+08				chr10:1348	N_Shore
cg26994413	1		37	12	1.31E+08	RIMBP2	NM_015347	Body	chr12:1309	S_Shore
cg27037109		1	37	19	12512034	ZNF799	NM_001080821	TSS200	chr19:1251	Island
cg27042491		2	37	10	77191686				chr10:7719	S_Shore
cg27062060		1	37	17	79258687	SLC38A10;SLC38A10	NM_001037984;NM_138570	Body;Body	chr17:7925	N_Shore
cg27072218	1		37	13	1.07E+08					
cg27101157	1		37	17	62340122	TEX2	NM_018469	5'UTR	chr17:6233	Island
cg27140170		1	37	2	2.31E+08	SP100;SP100	NM_001080391;NM_003113	TSS1500;TSS1500		
cg27181253		2	37	1	1.13E+08	WNT2B;WNT2B;WNT2B	NM_004185;NM_024494;NM_024494	Body;1stExon;5'UTR	chr1:11305	Island
cg27207308		1	37	20	47935683				chr20:4793	Island
cg27313674		1	37	20	6035120	LRRN4	NM_152611	TSS1500	chr20:6032	S_Shore
cg27323009		1	37	5	1.1E+08	TSLP;TSLP	NM_138551;NM_033035	TSS200;Body	chr5:11040	Island
cg27343123	1		37	2	62423136	B3GNT2	NM_006577	TSS200	chr2:62422	Island
cg27391127	1		37	10	1.04E+08	TRIM8	NM_030912	Body	chr10:1044	N_Shore
cg27426287		1	37	10	30025782	SVIL	NM_003174	TSS1500	chr10:3002	Island
cg27445005	1		37	18	76190006				chr18:7618	Island
cg27445005		2	37	18	76190006				chr18:7618	Island
cg27553486	1		37	7	930964	C7orf20	NM_015949	Body	chr7:93091	Island
cg27633010	1		37	10	1.35E+08	TUBGCP2	NM_006659	Body	chr10:1350	N_Shore
cg27633010		3	37	10	1.35E+08	TUBGCP2	NM_006659	Body	chr10:1350	N_Shore
cg27640020	1		37	19	19002253	GDF1;LASS1;LASS1	NM_001492;NM_198207;NM_021267	5'UTR;Body;Body	chr19:1900	N_Shelf
ch.16.1667936F		1	36	16	73947777					
ch.2.3048096R	1		36	2	1.48E+08					

Supplementary Document 1: Case and control eligibility and Selection Process

1. Subject Inclusion:

1.1. A subject with one or two knees with symptomatic OA that are eligible to be a Fast Progressor and/or a Non Progressor**.

An eligible knee is defined as:

1.1a. Mild-moderate symptomatic OA at baseline: in the same knee, KLG 2-3 and frequent pain (pain on most days of a month in past year) at baseline.

1.1b. Medial compartment JSW >= 1.5mm (V00MCMJSW >= 1.5) at baseline since at <1.5mm knee is already close to endstage and further fast progression is less unlikely to be preventable at that point; in addition JSW measurement is less reliable when knee approaches bone on bone.

1.1c. JSN in medial compartment at baseline is equal to or worse than JSN in lateral compartment

1.1d. JSW data at BL, 24m and 48m timepoints. (Exception: Fast Progressor knees can be missing JSW due to a TKR at 36 or 48m.)

1.1e. Adequate radiographic positioning at BL, 24 and 48m timepoints defined as VxxTPCFDS < 7.0mm and change in VxxTPCFDS < 2mm in magnitude BL to 24m or BL to 48m.

1.2. Subjects with only one eligible knee at baseline**:

1.2a. The contralateral knee KLG is less than or equal to the eligible knee at baseline. (E.g. Subjects with a KLG 2 symptomatic eligible knee can have a KLG 0-2 symptomatic or nonsymtomatic . If the eligible knee is KLG 3, then the contralataeral knee can be a KLG 0-3 symptomatic or nonysmptomatic knee. The contralateral knee must have a baseline medial JSW of >=1.5mm.

2.Subject Exclusion

2.1 TKR in either knee at BL, 12m or 24m.

2.2 RA diagnosis at any visit thru 72m (RAVISIT is 0,1,2,5,6 or 8). What about subjects with self-reported psoriatric arthritis, AS, or reactive arthritis? How many are there?

2.3 Oral Glucocorticoid use reported at >=2 visits from BL to 72m. How many are there?

3. Classify eligible knees of eligible subjects as Fast Progressor or Non-Progressor Index knees

3.1 Fast Progressor Index knees: JSW loss of more than 0.7mm between BL and 24m and remains narrowed more than 0.7mm at 48m or has a TKR by 48m.

3.2 Non-Progressor Index knees: JSW loss less than 0.5mm between BL and 24m and remains narrowed less than 0.5mm at 48m. Knee does not have TKR at 36m or 48m and does not have lateral JSN progression through 48m.

4. Fast Progressor Subjects

A Fast Progressor subject has one or two Fast Progressor Index knees.

For those with one Fast Progressor Index knee the contralateral knee can be:

4.1 A Non Progressor Index knee with a KLG ≤ to the KLG of the progressor knee;

4.2 An ineligible knee (e.g. KLG 0-1, KLG 2-3 nonsymptomatic and KLG ≤ to the KLG of Progressor knee) that meets Fast Progressor JSW criteria;

4.3 An ineligible knee that does not meet Fast Progressor criteria and KLG ≤ to the KLG of Progressor knee; or

4.4 An ineligible knee that does not meet conditions 1.1b to 1.1e and KLG ≤ to the KLG of Progressor knee.

5. Non Progressor Subjects

A Non Progressor Subject has one or two Non Progressor Index knees.

If a subject has only one Non Progressor Index knee:

5.1. The contralateral knee cannot be a Fast Progressor Index knee;

5.2. If the contralateral knee is not an eligible knee, then the contralateral knee must a) have KLG ≤ to the KLG of Progressor knee, b) allow assessment for Fast Progression (meet conditions 1.1b. to 1.1e.) and c) have JSW loss less than 0.5mm between BL and 24m and remain narrowed less than 0.5mm at 48m, d) does not have lateral JSN progression by 48m and e) does not have TKR at 36m or 48m.
